# A Positive Feedback Loop Links Opposing Functions of P-TEFb/Cdk9 and Histone H2B Ubiquitylation to Regulate Transcript Elongation in Fission Yeast

**DOI:** 10.1371/journal.pgen.1002822

**Published:** 2012-08-02

**Authors:** Miriam Sansó, Karen M. Lee, Laia Viladevall, Pierre-Étienne Jacques, Viviane Pagé, Stephen Nagy, Ariane Racine, Courtney V. St. Amour, Chao Zhang, Kevan M. Shokat, Beate Schwer, François Robert, Robert P. Fisher, Jason C. Tanny

**Affiliations:** 1 Department of Structural and Chemical Biology, Mount Sinai School of Medicine, New York, New York, United States of America; 2 Institut de Recherches Cliniques de Montréal, Montréal, Canada; 3 Department of Pharmacology and Therapeutics, McGill University, Montréal, Canada; 4 Programs in Biochemistry, Cell and Molecular Biology, Weill Cornell Medical College, New York, New York, United States of America; 5 Howard Hughes Medical Institute and Department of Cellular and Molecular Pharmacology, University of California San Francisco, San Francisco, California, United States of America; 6 Department of Microbiology and Immunology, Weill Cornell Medical College, New York, New York, United States of America; Albert Einstein College of Medicine, United States of America

## Abstract

Transcript elongation by RNA polymerase II (RNAPII) is accompanied by conserved patterns of histone modification. Whereas histone modifications have established roles in transcription initiation, their functions during elongation are not understood. Mono-ubiquitylation of histone H2B (H2Bub1) plays a key role in coordinating co-transcriptional histone modification by promoting site-specific methylation of histone H3. H2Bub1 also regulates gene expression through an unidentified, methylation-independent mechanism. Here we reveal bidirectional communication between H2Bub1 and Cdk9, the ortholog of metazoan positive transcription elongation factor b (P-TEFb), in the fission yeast *Schizosaccharomyces pombe*. Chemical and classical genetic analyses indicate that lowering Cdk9 activity or preventing phosphorylation of its substrate, the transcription processivity factor Spt5, reduces H2Bub1 in vivo. Conversely, mutations in the H2Bub1 pathway impair Cdk9 recruitment to chromatin and decrease Spt5 phosphorylation. Moreover, an Spt5 phosphorylation-site mutation, combined with deletion of the histone H3 Lys4 methyltransferase Set1, phenocopies morphologic and growth defects due to H2Bub1 loss, suggesting independent, partially redundant roles for Cdk9 and Set1 downstream of H2Bub1. Surprisingly, mutation of the histone H2B ubiquitin-acceptor residue relaxes the Cdk9 activity requirement in vivo, and *cdk9* mutations suppress cell-morphology defects in H2Bub1-deficient strains. Genome-wide analyses by chromatin immunoprecipitation also demonstrate opposing effects of Cdk9 and H2Bub1 on distribution of transcribing RNAPII. Therefore, whereas mutual dependence of H2Bub1 and Spt5 phosphorylation indicates positive feedback, mutual suppression by *cdk9* and H2Bub1-pathway mutations suggests antagonistic functions that must be kept in balance to regulate elongation. Loss of H2Bub1 disrupts that balance and leads to deranged gene expression and aberrant cell morphologies, revealing a novel function of a conserved, co-transcriptional histone modification.

## Author Summary

Modification of histone proteins is an important transcriptional regulatory mechanism in eukaryotic cells. Although various histone modifications are found primarily within the coding regions of transcribed genes, how they influence transcription elongation remains unclear. Among these modifications is mono-ubiquitylation of histone H2B (H2Bub1), which is needed for co-transcriptional methylation of histone H3 at specific sites. Here we show that H2Bub1 and Cdk9, the kinase component of positive transcription elongation factor b (P-TEFb), are jointly regulated by a positive feedback loop: Cdk9 activity is needed for co-transcriptional H2Bub1, and H2Bub1 in turn stimulates Cdk9 activity toward one of its major substrates, the conserved elongation factor Spt5. We provide genetic evidence that the combined action of H2Bub1 on Spt5 phosphorylation and histone methylation accounts for the gene-regulatory effects of this modification. Surprisingly, our genetic and genome-wide studies indicate that P-TEFb and H2Bub1 act in *opposition* on elongating RNA polymerase. We suggest that the positive feedback linking P-TEFb and H2Bub1 helps to maintain a balance between their opposing actions. These results highlight a novel regulatory role for a conserved histone modification during transcription elongation.

## Introduction

The elongation phase of transcription is a point of regulation for many genes transcribed by RNAPII in eukaryotes, and control of elongation is critical for coupling of transcription to downstream steps in gene expression [1], [2]. Whereas the regulation of transcription at the initiation step has been studied extensively, many of the mechanisms governing elongation in vivo remain to be elucidated. Transcription is accompanied by post-translational modification of nucleosomal histones in a highly conserved pattern, stereotypical features of which include methylation of histone H3 Lys4 (H3K4me) and acetylation of histones H3 and H4 at 5′ ends, methylation of histone H3 Lys36 (H3K36me) towards 3′ ends, and mono-ubiquitylation of histone H2B at a conserved site in the carboxyl-terminus (H2Bub1) throughout coding regions of genes [3]. Conservation of this pattern suggests an important role in coordinating gene expression, but the precise functions of individual modifications in elongation control are poorly understood.

H2Bub1, which appears to play a central role in the interplay between chromatin and the RNAPII elongation complex, is catalyzed in budding yeast by the ubiquitin-conjugating enzyme Rad6 and the E3 ubiquitin ligase Bre1 [4]. Formation of H2Bub1 on transcribed chromatin also requires PAF, a conserved complex with multiple functions during elongation [5]. H2Bub1 is required for co-transcriptional generation of H3K4me by the methyltransferase Set1 in yeast, and contributes to global H3K4me levels in metazoans [6]–[9]. In vitro, H2Bub1 directly stimulates activity of Set1 towards a reconstituted chromatin substrate [10], [11].

H2Bub1 also acts independently of histone methylation; our work in the fission yeast *S. pombe* revealed that the Set1-independent pathway is important for normal cell growth and morphology, and for elongation by RNAPII at select target genes [12]. Similar findings have now been reported in *S. cerevisiae* and mammalian cells [13]–[15], but the mechanism of methylation-independent effects of H2Bub1 is unknown.

Co-transcriptional histone modifications are regulated by a conserved subset of cyclin-dependent kinases (CDKs) associated with the transcription machinery [16]. In metazoans these include Cdk7, a component of the initiation factor TFIIH, and Cdk9, catalytic subunit of positive transcription elongation factor b (P-TEFb). Cdk7, Cdk9 and their yeast orthologs phosphorylate multiple proteins important for elongation, including the Rpb1 subunit of RNAPII, at Ser2, Ser5 and Ser7 positions within the repeated YSPTSPS motif of its carboxyl-terminal domain (CTD) [17]–[19]; and the Spt5 subunit of a conserved elongation factor, known in metazoans as DRB-sensitivity inducing factor (DSIF) [20], [21]. Those phosphorylations control recruitment of pre-mRNA-processing and chromatin-modifying enzymes [17], [22], [23].In metazoans, moreover, Cdk9 activity overcomes promoter-proximal pausing imposed by unphosphorylated DSIF and a negative elongation factor (NELF) [20], [24], [25]. Pausing is thought to act both as a quality control over gene expression, by facilitating recruitment of mRNA-processing factors [26], [27]; and as a rate-limiting determinant of expression for subsets of stringently regulated genes [28], [29].

P-TEFb also regulates histone modification. In mammalian cells, levels of H2Bub1, H3K4me, and H3K36me decrease after depletion of Cdk9 by RNA interference (RNAi), or treatment with flavopiridol, an inhibitor of Cdk9 and related kinases [30], [31]. Bur1, the essential ortholog of Cdk9 in budding yeast, is required for co-transcriptional H3K36me and consequent action of the Rpd3S histone deacetylase complex to prevent transcription initiation within coding regions [32]–[34]. Similarly, co-transcriptional H2Bub1 depends on Bur1 [35], [36] and the carboxyl-terminal region of Spt5, which contains sites phosphorylated by Bur1 [37], [38]. Budding yeast contains another, non-essential CDK, Ctk1, which is the major Rpb1-Ser2 kinase in vivo and is required for H3K36me [39], [40]. Although Ctk1 was proposed to be a yeast-specific P-TEFb paralog [41], putative orthologs of Ctk1 have recently been identified in human and *Drosophila* cells [42], [43], suggesting faithful conservation of the entire CTD kinase network.

CDKs are themselves regulated by histone modification. In mammals, P-TEFb binds the bromodomain protein Brd4, whose recruitment to promoter-proximal sites is stimulated by acetylation of histone H4 [44], [45]. Phosphorylation of histone H3 also favors recruitment of P-TEFb [46], [47]. On the other hand, certain histone marks work in opposition to specific CDKs during elongation. For example, in budding yeast, lethality of a *BUR1* deletion is suppressed by deletion of the H3K36 methyltransferase Set2 [34], [48]; and H2B de-ubiquitylation by Ubp8—a component of the SAGA complex—is required for Ctk1 recruitment and Ser2 phosphorylation at some genes [49].

In fission yeast, the P-TEFb ortholog is the essential Cdk9/Pch1 complex. In vitro, Cdk9 phosphorylates the Rpb1 CTD at both Ser5 and Ser2 [50]; inhibition of Cdk9 in vivo reduced the apparent stoichiometry of Rpb1 phosphorylation but did not cause selective loss of either Ser5 or Ser2 signals [51]. Cdk9 also phosphorylates the CTD of *S. pombe* Spt5 [52]; *spt5* mutations that prevent this phosphorylation cause slow growth, defects in transcript elongation and, when combined with partial truncations of the Rpb1 CTD, synthetic lethality [53]. Genetic epistasis suggests, moreover, that the Spt5 CTD contains an exclusive, albeit nonessential, target of Cdk9 in vivo [51]. The potential roles of CDKs and their substrates in regulating histone modifications in fission yeast have not been explored.

Here we uncover a mutual dependence of H2Bub1 and P-TEFb function in *S. pombe*: mutations that impair phosphorylation of Spt5 by Cdk9 diminish levels of H2Bub1 and, reciprocally, mutants unable to generate H2Bub1 have reductions in chromatin-associated Cdk9 and phosphorylated Spt5. Ablation of the preferred phosphoacceptor site in Spt5 combined with deletion of *set1^+^* phenocopies morphological defects of *htb1-K119R* mutants, suggesting that Cdk9 and Set1 govern separate, partially redundant pathways downstream of H2Bub1. Despite the dependencies between H2Bub1 and Spt5 phosphorylation, mutations that impair Cdk9 function suppress abnormal cell morphologies and reverse an RNAPII distribution defect, both due to loss of H2Bub1. Conversely, growth of *htb1-K119R* cells is resistant to selective Cdk9 inhibition, relative to that of *htb1^+^* cells. Taken together, biochemical and genetic results suggest that P-TEFb and H2Bub1 oppose one another to regulate RNAPII elongation, but that proper balance between the two is ensured via a positive feedback loop, in which phosphorylation by Cdk9 stimulates H2Bub1 and vice versa.

## Results

### H2Bub1 influences global RNAPII distribution in *S. pombe*

**Figure 1:**
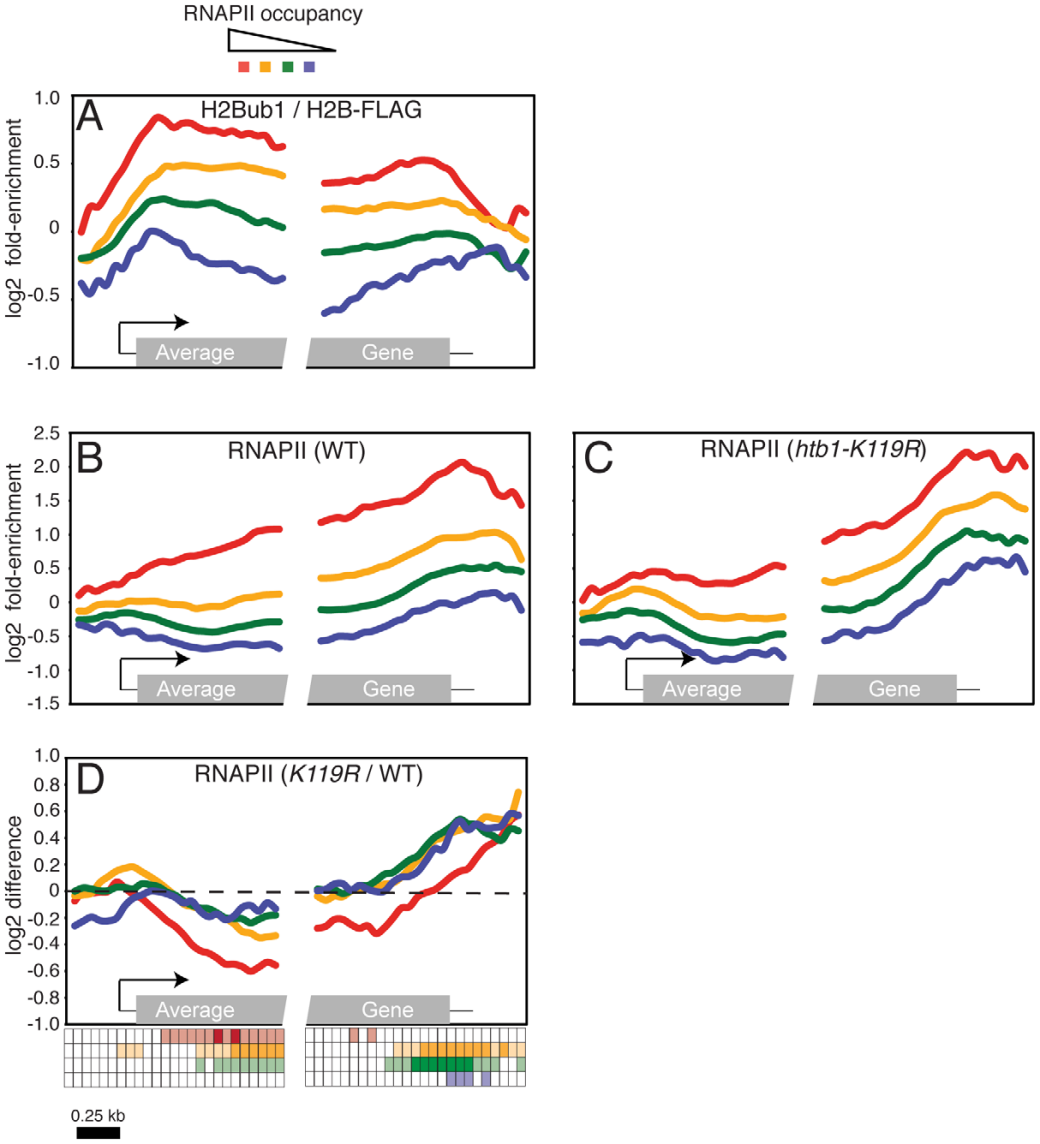
Loss of H2Bub1 globally alters distribution of RNAPII in gene coding regions. (A) Average distribution of H2Bub1 at 540 *S. pombe* genes, as determined by ChIP-chip. Genes were grouped according to total levels of RNAPII enrichment (see key at top). The grey box in the “average gene” representation at bottom denotes the gene coding region; 5′ and 3′ untranslated regions are denoted by thin black lines. The arrow denotes the transcription start site. (B) Average distribution of RNAPII at 540 *S. pombe* genes, as determined by ChIP-chip in a wild-type strain (JTB62-1). Genes were grouped according to total levels of RNAPII enrichment. (C) As in (B), determined in an *htb1-K119R* mutant strain (JTB67-1). Gene groupings were created using wild-type RNAPII enrichment values. (D) Average distributions of differences between mutant and wild-type RNAPII enrichment grouped according to RNAPII enrichment in wild-type cells. The key below the graph illustrates the statistical significance of the differences for each group at 50 positions along the average gene. The rows of the key are color-coded according to the graph. Open squares denote p>0.01; light shading denotes 0.01>p>10exp-5; dark shading denotes p<10exp-5 (one-sample t-tests; μ_0_ = 0). Note that there is only light shading for the last row (corresponding to the blue curve).

Our previous results suggested that H2Bub1 promotes expression of a subset of genes [12]. To ask whether H2Bub1 might act more generally in transcription, we combined ChIP with hybridization of recovered DNA sequences to microarrays that cover the entire *S. pombe* genome at 200 base-pair intervals (ChIP-chip). We first mapped genome-wide distribution of H2Bub1 in wild-type cells with an antibody that specifically recognizes the ubiquitylated form of histone H2B [[54], Figure S1A]. A FLAG-epitope tag fused to the carboxyl-terminus of histone H2B allowed us to normalize for total H2B occupancy by parallel ChIP-chip with anti-FLAG antibody. The FLAG-tagged H2B was ubiquitylated to a similar extent as the native protein ([12], Figure S1B). To correlate the H2Bub1 pattern with transcription, we also performed ChIP-chip with an RNAPII-specific antibody. We grouped genes into quartiles according to their total levels of RNAPII enrichment and plotted average distributions of H2Bub1 within each group (Figure 1A). This analysis confirmed the presence of H2Bub1 throughout coding regions of transcribed genes in *S. pombe*, similar to its distribution in *S. cerevisiae* and metazoans [13], [55]–[57]. Furthermore, H2Bub1 enrichment was correlated with that of RNAPII throughout the genome (*r* = 0.58).

Because of this genome-wide association, we sought to determine the impact of H2Bub1 loss on RNAPII distribution within genes, by ChIP-chip in wild-type and *htb1-K119R* mutant cells. This analysis revealed alterations in RNAPII occupancy within gene coding regions in the *htb1-K119R* mutant: a global, downstream shift in average RNAPII density, reflecting both a decrease in occupancy within the 5′ halves of genes and a more pronounced increase near the 3′ ends (Figure 1B and 1C). These changes occurred in all classes of genes, but varied quantitatively depending on their overall levels of RNAPII cross-linking (Figure 1D; Dataset S1). Genes highly enriched for RNAPII (Figure 1D; red line) had a significant (*P*<10exp-5) decrease in RNAPII 5′-end occupancy in the absence of H2Bub1, whereas the increase at 3′ ends failed to reach significance (*P*>0.01). In the other three classes of genes the increases in RNAPII occupancy at the 3′ end were the most significant changes (Figure 1D). Therefore, H2Bub1 globally affects RNAPII distribution in *S. pombe* genes.

Previous reports have shown that H2Bub1 affects nucleosome stability [9], [14], [58], [59]. We performed ChIP-chip with an antibody that recognizes histone H3 to determine whether the changes in RNAPII distribution caused by H2Bub1 loss were correlated with changes in nucleosome occupancy. The average histone H3 occupancy in gene coding regions did not differ significantly between wild-type and *htb1-K119R* cells (Figure S2; Datasets S2 and S3). These data suggest that the observed changes in RNAPII occupancy are unlikely to be caused solely by altered nucleosome distribution.

To ascertain whether genes affected by the *htb1-K119R* mutation were associated with particular cellular functions, we searched for Gene Ontology (GO) terms that were significantly enriched among the 500 genes with the largest increases or decreases in RNAPII occupancy in the *htb1-K119R* mutant. The genes exhibiting loss of RNAPII occupancy were enriched for ribosomal protein genes (*P* = 3.58exp-26, 17.7%), consistent with a role for H2Bub1 in promoting RNAPII occupancy at heavily transcribed genes.

The ChIP-chip analysis revealed that RNAPII density was reduced throughout coding regions of 25 of 40 genes whose expression decreased by ≥2-fold in our previous microarray analysis of gene expression [12](Table S1). Furthermore, this group of genes is significantly enriched among the highest RNAPII-occupancy class that we defined by ChIP-chip (*P* = 7.96exp-5). Thus, the ChIP-chip results and our previous gene-expression data argue that loss of H2Bub1 has a particularly strong negative impact on steady-state levels of poly(A^+^) mRNA produced from highly transcribed genes. However, the global redistribution of RNAPII in *htb1-K119R* cells (Figure 1D) suggests that H2Bub1 might affect gene expression more broadly through additional, post-transcriptional mechanisms.

### P-TEFb activity promotes H2Bub1 in fission yeast

**Figure 2:**
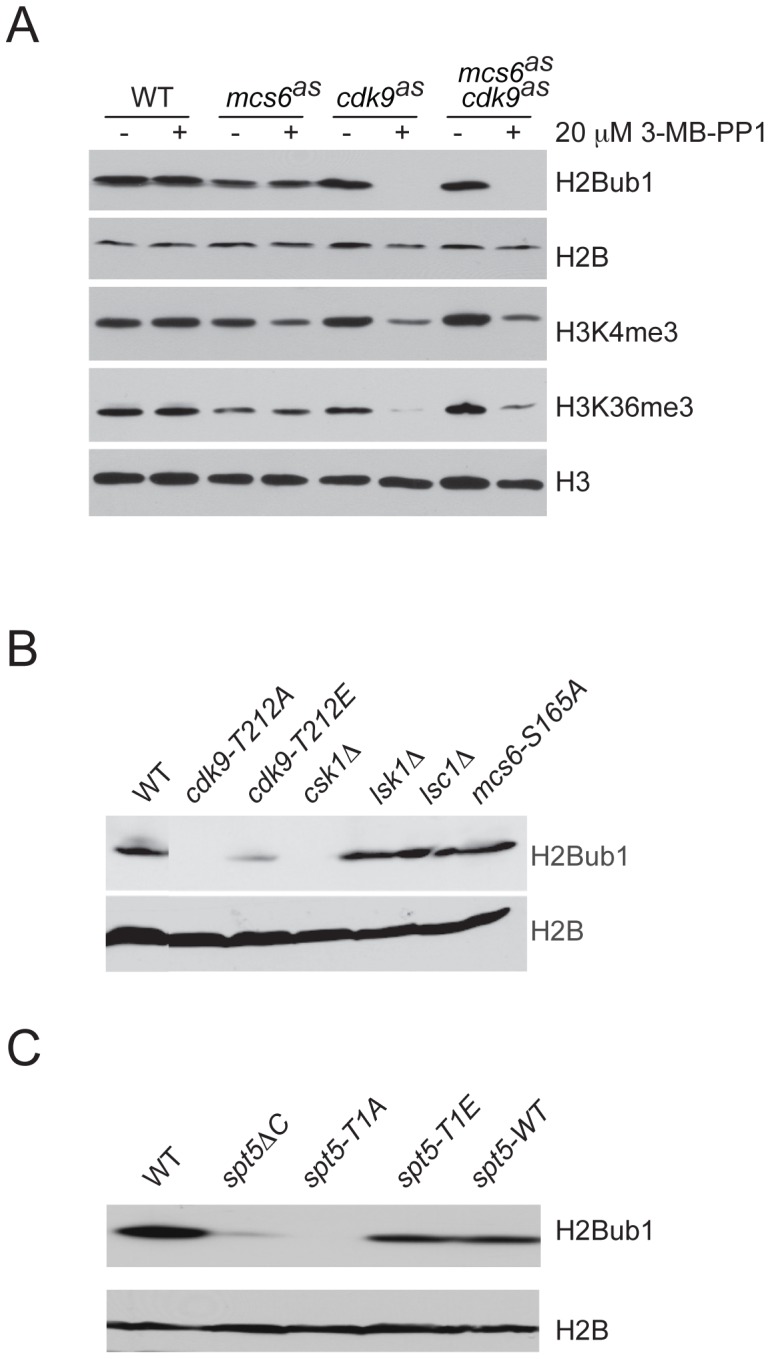
H2Bub1 depends on Cdk9 activity and Spt5 phosphorylation. (A) Immunoblots of whole-cell extracts from wild-type (wt) (JS78) or AS mutant strains (LV7, LV77, LV42), as indicated, grown in the absence (−) or presence (+) of 20 µM 3-MB-PP1, added 20 min prior to harvest. Antibodies are indicated at right. (B) Immunoblots of extracts from indicated strains probed for total histone H2B and H2Bub1, as indicated at right. “T212A” and “T212E” denote strains *cdk9-T212A* (HD7-24) and *cdk9-T212E* (HG127). (C) Immunoblots of extracts from wild-type (JS78) or indicated *spt5* mutant strains.

Loss of H2Bub1 caused changes in cell morphology and RNAPII distribution that resemble those produced by inactivation of Rpb1 CTD kinases or loss of Rpb1-CTD Ser2 phosphorylation [12], [51], [60], [61]. We therefore investigated possible interactions between H2Bub1 and three CDKs implicated in transcription: the TFIIH-associated Cdk7 ortholog Mcs6 [61], Cdk9, and the Ctk1 ortholog Lsk1 [62]. First, we determined the effect on H2Bub1 of selective inhibition of Mcs6 or Cdk9, made possible by mutating the “gatekeeper” residue in each kinase to Gly, to render the enzyme sensitive to bulky adenine analogs that do not affect wild-type kinases [63]. We previously replaced *mcs6^+^*, *cdk9^+^* and *lsk1^+^* with alleles encoding analog-sensitive (AS) mutant versions, each of which was sensitive to the inhibitory analog 3-MB-PP1 [51]. Selective inhibition of Cdk9, by addition of 20 µM 3-MB-PP1 to *cdk9^as^* cells, caused diminution of H2Bub1 signals in whole-cell extracts (Figure 2A). Cdk9 inhibition also decreased levels of H3K4 di- and trimethylation (H3K4me2 and H3K4me3, respectively) and H3K36 trimethylation (H3K36me3) (Figure 2A and data not shown)—modifications likewise associated with actively transcribed genes [3]. Inhibition of the TFIIH-associated kinase in *mcs6^as^* cells had small or no effects on H2Bub1, H3K4me2, H3K4me3 or H3K36me3. These results suggest a predominant role for Cdk9 in co-transcriptional histone modification, and a possibly exclusive requirement in generating H2Bub1.

Bur1 in budding yeast and Cdk9 in mammalian cells are proposed to stimulate H2Bub1 by favoring recruitment of PAF, which can in turn associate directly with the H2B ubiquitylation machinery [10], [37], [38], [64]. Inhibition of Cdk9^as^ decreased association of both the PAF component Rtf1 and the E2 ubiquitin-conjugating enzyme Rhp6 with transcribed chromatin (Figure S3A and S3B), suggesting faithful conservation of the P-TEFb-H2Bub1 pathway in *S. pombe*.

### H2Bub1 depends on activated Cdk9 and phosphorylation of Spt5

To further probe the dependence of H2Bub1 on P-TEFb, we tested effects of other mutations that perturb Cdk9 function (Figure 2B). Two mutant strains in which Cdk9 activity is reduced by lack of phosphorylation at Thr212 of the activation segment (T loop)—*cdk9-T212A* and *csk1Δ*, which is missing the CDK-activating kinase (CAK) responsible for that phosphorylation [50]—had nearly undetectable levels of H2Bub1. In contrast, a *cdk9-T212E* mutation, which substitutes a Glu residue for Thr212 to mimic constitutive phosphorylation [65], had only a small negative effect on H2Bub1. Loss of H2Bub1 occurred uniquely in mutants with impaired Cdk9 function; neither *mcs6-S165A*, a T-loop mutation that renders Mcs6 refractory to CAK [66], [67], nor deletions of *lsk1^+^* or *lsc1^+^* [which encodes the cyclin partner of Lsk1 [68]], affected bulk H2Bub1 levels (Figure 2B). Therefore, among *S. pombe* CDKs implicated in transcript elongation, Cdk9 is uniquely required for H2Bub1.

Impaired Cdk9 function could lead to H2Bub1 loss by decreasing phosphorylation of the Spt5 CTD nonapeptide repeat TPAWNSGSK at Thr1, a site modified by Cdk9 in vitro [52]. To test this we measured H2Bub1 levels in a series of *spt5* truncation mutants: *spt5ΔC*, in which the entire CTD is deleted; or variants containing seven repeats, either of wild-type sequence (*spt5-WT*), or with every Thr1 position mutated to Ala (*spt5-T1A*) or Glu (*spt5-T1E*) [53]. H2Bub1 decreased in *spt5ΔC* and *spt5-T1A* mutants, but not in *spt5-WT* or an *spt5-T1E* mutant that mimics constitutive phosphorylation (Figure 2C). We conclude that Cdk9 activity and Spt5-CTD phosphorylation are required for H2Bub1 in vivo.

### Cdk9 activity is required for Spt5-Thr1 phosphorylation in vivo

**Figure 3:**
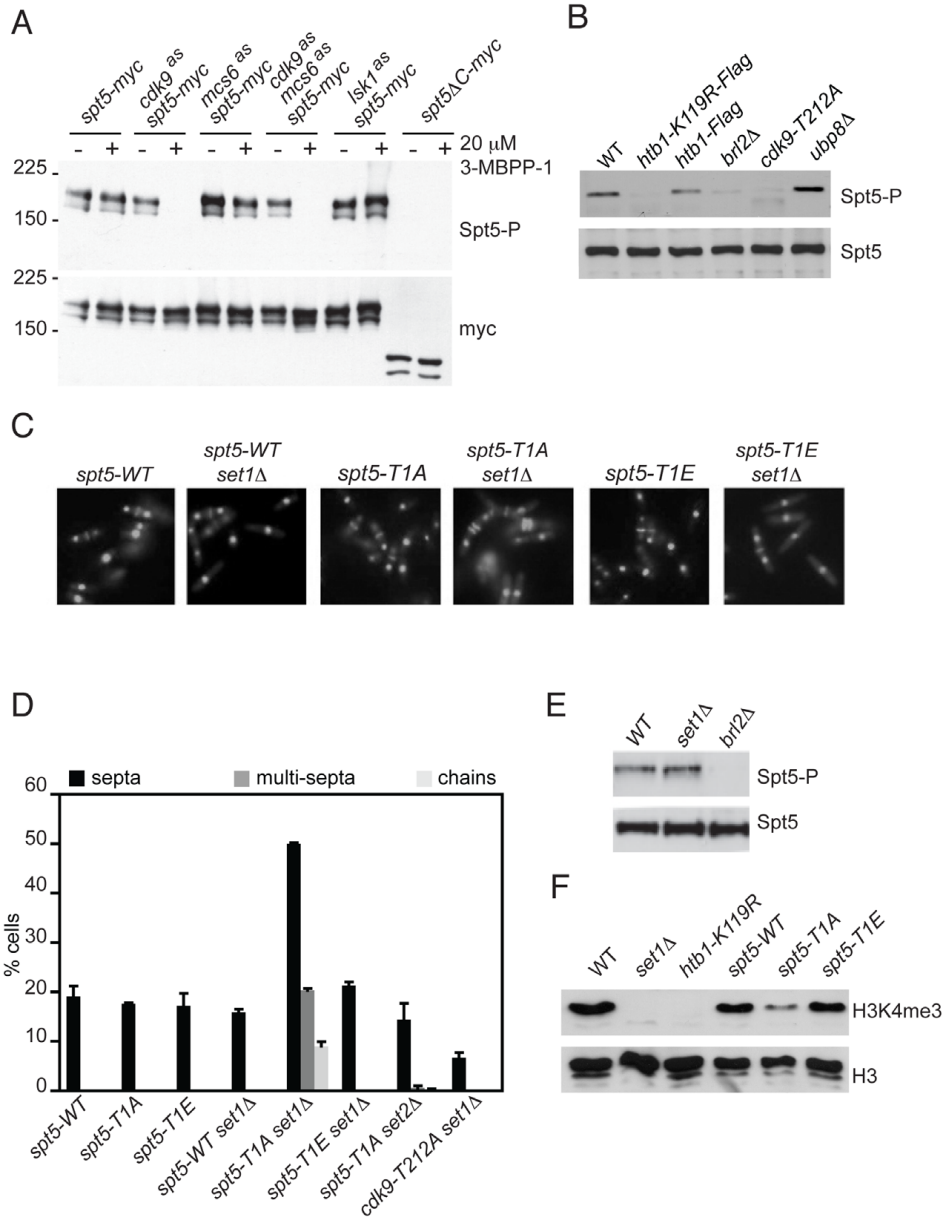
H2Bub1 independently stimulates Cdk9-mediated Spt5 phosphorylation and Set1-dependent H3K4 methylation. (A) Immunoblots of extracts from strains carrying *spt5*-*myc* with or without an *as* kinase allele as indicated (CS111, CS112, CS155, CS159, LV125 and LV167, respectively). Cultures were grown in the absence (−) or presence (+) of 20 µM 3-MB-PP1, added 20 min prior to harvest. Antibody reactivities are indicated at right. (B) Immunoblots of extracts from indicated strains (JS78, JTB67-1, JTB62-1, JTB331, HD7-24 and JTB297, respectively), probed for phosphorylated (Spt5-P) or total Spt5. (C) Fluorescent images of DAPI/calcofluor-stained cells from indicated strains (*spt5-WT*, JTB350, *spt5-T1A*, JTB352, *spt5-T1E*, JTB354, respectively). (D) Quantification of abnormal septation in strains of indicated genotypes (JTB67-1, *spt5-WT*, JTB350, *spt5-T1A*, JTB352, *spt5-T1E*, JTB354, JTB418 and JTB428, respectively). Error bars represent standard deviations from 2 independent experiments; at least 200 cells were counted in each. (E) Immunoblots of extracts from indicated strains (JTB204, JTB80-2, and JTB331, respectively), probed for phosphorylated (Spt5-P) or total Spt5. (F) Immunoblots of extracts from indicated strains (JTB204, JTB80-2, JTB67-1, *spt5-WT, spt5-T1A* and *spt5-T1E*, respectively), probed for H3K4me3 or total H3.

To investigate this connection, and to assess relative contributions of different CDKs to Spt5 phosphorylation in vivo, we generated an antibody specific for the Spt5 CTD phosphorylated on the Thr1 residue (anti-Spt5-T1P), which recognized Spt5 only after it had been phosphorylated by Cdk9 in vitro (Figure S4A). In whole-cell extracts, the phosphorylated Spt5 signal was undetectable after treatment of *cdk9^as^* cells with 20 µM 3-MB-PP1, which had little or no effect on Spt5 phosphorylation in wild-type, *mcs6^as^* or *lsk1^as^* cells, and did not alter Spt5 expression levels in any of the strains (Figure 3A). Spt5-Thr1 phosphorylation was nearly abolished in *cdk9^as^* cells at 3-MB-PP1 doses as low as 300 nM (Figure S4B). In contrast, the IC_50_ for inhibition of *cdk9^as^* cell growth was ∼10–15 µM, and Rpb1-CTD Ser2 phosphorylation was relatively resistant to inhibition of Cdk9 alone [51]. There was no additive sensitivity of Spt5-P to the analog in *mcs6^as^ cdk9^as^* cells, nor any loss of Spt5-P signal in *mcs6^as^* cells treated with 3-MB-PP1 doses up to 40 µM (Figure S4B, S4C). We conclude that Cdk9 is the major, and possibly sole, kinase responsible for phosphorylation of Spt5-Thr1 in vivo, and that levels of Spt5-P are sensitive even to sublethal reductions in Cdk9 activity.

### A role for Spt5 phosphorylation downstream of H2Bub1

There is a precedent for H2Bub1 regulating the function of a CTD kinase [49]. We wondered whether Cdk9 and the H2Bub1 machinery could be reciprocally regulated, such that the H2Bub1-defective mutants *htb1-K119R* and *brl2Δ* (which lacks the Bre1 ortholog required for H2Bub1 [12]) would have global alterations in Spt5-P. Indeed, Spt5-P was decreased relative to total Spt5 (detected with antibodies specific for wild-type Spt5-CTD irrespective of phosphorylation state; Figure S4D) in both *htb1-K119R* and *brl2Δ* strains. Conversely, Spt5-P was slightly increased in cells lacking the ubiquitin-specific protease encoded by *ubp8^+^* (Figure 3B). (Cells lacking a second putative H2B de-ubiquitylating enzyme, encoded by the *UBP10* homolog *ubp16^+^* [69], had no change in levels of H2Bub1 or Spt5-P [data not shown]). These data suggest a positive feedback loop connecting Cdk9 and the H2Bub1 machinery in vivo.

Previous reports have documented cooperative action of H2Bub1 and the FACT complex, a conserved elongation factor that controls nucleosome dynamics during RNAPII elongation [14], [70], [71]. This prompted us to ask whether FACT might also influence Spt5-P levels in vivo. There was no reduction in levels of Spt5-P, relative to total Spt5, in a strain deleted for *pob3^+^*, which encodes a subunit of FACT (Figure S5A). This argues that promotion of Spt5-P reflects a FACT-independent function of H2Bub1.

Another consequence of H2Bub1 loss is reduction in H3K4me—a chromatin modification strongly implicated in gene activation. We showed previously, however, that loss of H2Bub1 caused more severe phenotypes than did ablation of H3K4me due to deletion of *set1^+^*. Those phenotypes include increased frequency of cells with division septa in asynchronous populations, and the appearance of cells with abnormal septation patterns (multiple septa separating two nuclei and unseparated chains of cells in which septa had formed) [12]. To test whether impaired Spt5-Thr1 phosphorylation might account for the more severe phenotypes caused by H2Bub1 loss, relative to those due to absence of H3K4me, we constructed an *spt5-T1A set1Δ* double mutant; the combination, which abolished two protein modifications shown to depend on H2Bub1, phenocopied the increased and aberrant septation produced by H2Bub1 loss (Figure 3C, 3D). Unlike the *htb1-K119R* and *brl2Δ* mutants, a *set1Δ* mutant showed no reduction in Spt5 phosphorylation (Figure 3E). Conversely, the *spt5-T1A* mutant retained detectable levels of H3K4me3 despite reduced H2Bub1 (Figure 3F). Therefore, H2Bub1 promotes Spt5-P and H3K4me by independent pathways. Importantly, the *spt5-T1A* mutation by itself did not cause morphological defects, even though it lowered H2Bub1 levels. We attribute this to the residual Set1 function present in the *spt5-T1A* strain (Figure 3F). A similar double mutant carrying *spt5-T1E* was morphologically normal, as was an *spt5-T1A set2Δ* strain, confirming the specificity of the genetic interaction (Figure 3C, 3D). (The *pob3Δ* mutation had no effect on cell morphology by itself, and did not modify phenotypes caused by *htb1-K119R*, suggesting that these effects are also FACT-independent [Figure S5B].) Together, these results suggest that Cdk9 and Set1 operate downstream of H2Bub1 to modify components of the transcription machinery and chromatin, respectively, and thereby regulate transcript elongation.

Next, to investigate the basis for reduced Spt5-P in H2Bub1-deficient cells, we measured Spt5-P occupancy at specific genes in the *htb1-K119R* mutant by ChIP. Control experiments confirmed that the Spt5-P antibody was suitable for immunoprecipitation and ChIP (Figure S6A, S6B). As predicted by immunoblot results, treatment of *cdk9^as^ spt5-myc* cells with 3-MB-PP1 abolished the Spt5-P ChIP signal without affecting total Spt5 recruitment.

**Figure 4:**
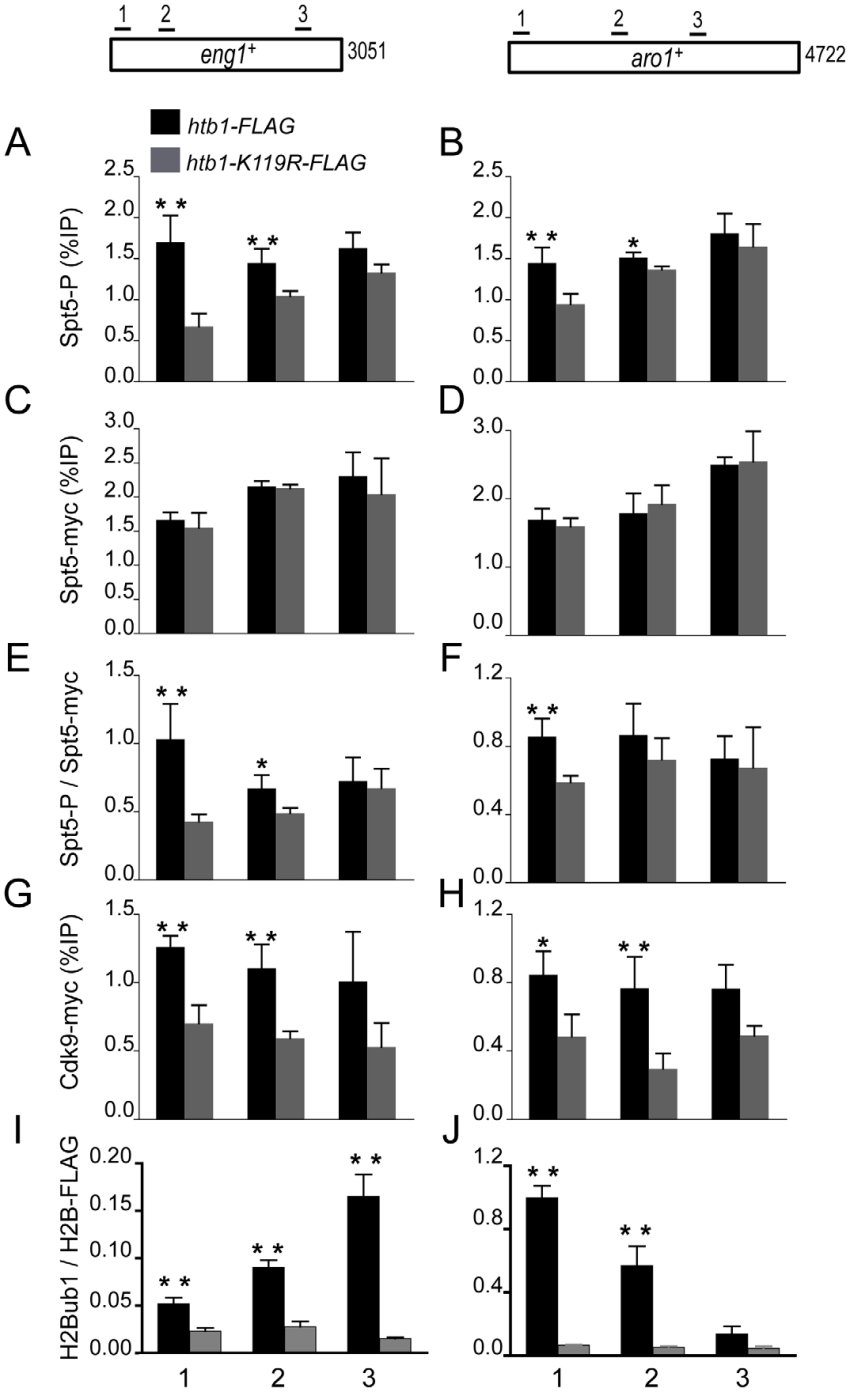
H2Bub1 enhances Cdk9 recruitment and Spt5 phosphorylation at transcribed genes. (A,B) Spt5 phosphorylation was measured by ChIP using anti-Spt5-P in *spt5-myc* (MS265; black bars) and *spt5-myc htb1-K119R* (LV239; gray bars) strains and quantified at the indicated genes by qPCR. Enrichment is plotted as a percentage of input signal for each primer pair. Positions of PCR primer pairs within coding regions are indicated schematically at top. (C,D) Spt5-myc occupancy was measured by ChIP as in A and B. (E,F) Spt5-P enrichment normalized to total Spt5-myc occupancy. (G,H) Cdk9-myc occupancy was measured by ChIP in *cdk9-myc* (MS264; black bars) and *cdk9-myc htb1-K119R* (KL259; gray bars) strains. (I,J) H2Bub1 enrichment was measured by ChIP and normalized to H2B-FLAG occupancy. Error bars denote standard deviations from 3 independent experiments. Asterisks denote a significant difference between wild-type and mutant (“*” p<0.04, “**” p<0.02; unpaired t-test).

We then compared cross-linking of Spt5-P to chromatin in *htb1^+^* and *htb1-K119R* cells, at the Cdk9-sensitive *eng1^+^* and Cdk9-insensitive *aro1^+^* genes [51] (Figure 4A and 4B); and at two loci (*nup189^+^* and *SPBC354.10^+^*) that, according to our RNAPII ChIP-chip analysis, displayed a 3′ shift in RNAPII distribution in the absence of H2Bub1 (Figure S7A, S7B). A non-transcribed sequence served as a negative control (Figure S8). Although Spt5-P was broadly enriched within gene coding regions, as we observed for H2Bub1, the patterns of Spt5-P and H2Bub1 crosslinking across *eng1^+^* and *aro1^+^* differed, suggesting that the interdependence between these modifications is influenced by other, potentially locus-specific factors (Figure 4A, 4B, 4I, and 4J). Nevertheless, levels of Spt5-P were reduced to varying degrees on all four genes in the *htb1-K119R* mutant (Figure 4A and 4B, Figure S7A and S7B). Total Spt5 crosslinking was unchanged in *htb1-K119R* relative to wild-type cells, and the effect on Spt5-P was most evident near the 5′ ends of genes we tested, suggesting that H2Bub1 promotes co-transcriptional phosphorylation (but not recruitment) of Spt5 during the transition from initiation to elongation (Figure 4C–4F; Figure S7C–S7F).

We hypothesized that the reduction in Spt5-P could be due to a defect in recruitment of Cdk9 to chromatin in the *htb1-K119R* mutant. To test this possibility we compared Cdk9 crosslinking to *eng1^+^*, *aro1^+^*, *nup189^+^* and *SPBC354.10^+^* by ChIP in *htb1^+^ cdk9-myc* and *htb1-K119R cdk9-myc* strains (Figure 4G and 4H, Figure S7G and S7H). This analysis revealed that Cdk9 recruitment was impaired at all four loci in *htb1-K119R* cells, consistent with the reduction in Spt5-P signals. Diminished Spt5-P and Cdk9 crosslinking to these loci was not an indirect consequence of reduced RNAPII recruitment, because RNAPII crosslinking and mRNA levels were not reduced at three of the four genes examined (Figure S9). We conclude that lack of H2Bub1 specifically impedes Cdk9 recruitment to transcribed chromatin, leading to impaired phosphorylation of Spt5 within gene coding regions.

### Mutual genetic suppression by P-TEFb and H2Bub1 pathway mutations

**Figure 5:**
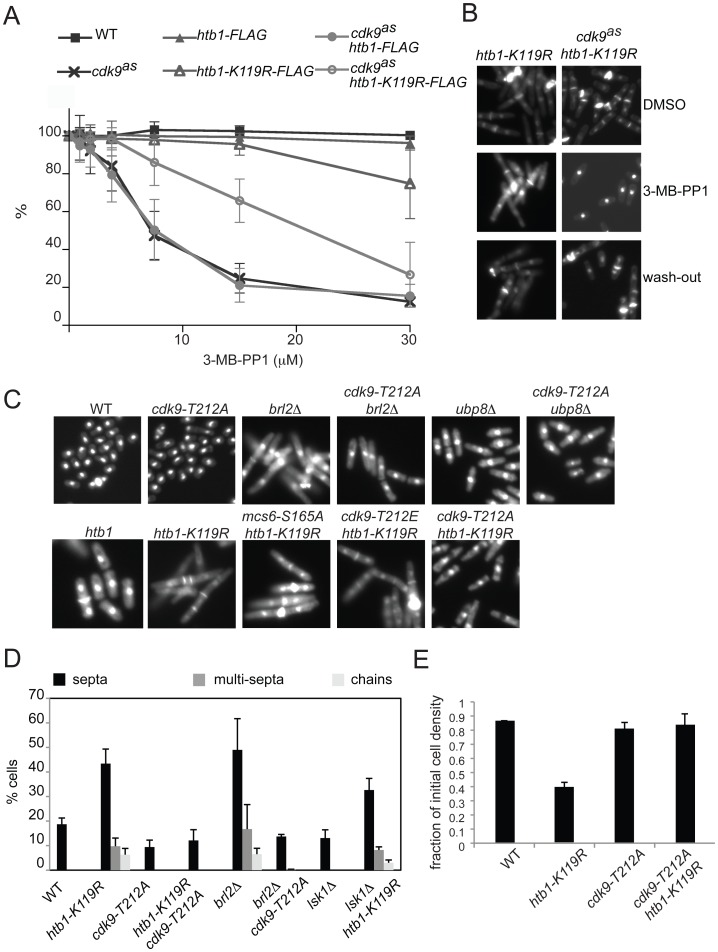
Mutual phenotypic suppression in *cdk9 htb1-K119R* double mutants. (A) For indicated strains (JS78, LV7, JTB62-1, JTB67-1, MS249, LV193), growth in increasing [3-MB-PP1] is plotted as a percentage of growth in the absence of 3-MB-PP1. Error bars denote standard deviations from 3 independent experiments. (B) Images of DAPI- and calcofluor-stained *htb1-K119R* (JTB67-1) and *cdk9^as^ htb1-K119R* (LV193) cells grown in the absence (top) or presence (middle) of 10 µM 3-MB-PP1 for 7 hr, or after inhibitor washout and return to growth (bottom). (C) Fluorescent images of DAPI/calcofluor-stained wild-type (JS78), *cdk9-T212A* (HD7-24), *brl2Δ* (JTB331), *brl2Δ cdk9-T212A* (JTB335), *ubp8Δ* (JTB297), *ubp8Δ cdk9-T212A* (JTB336), *htb1-K119R* (JTB67-1), *cdk9-T212A htb1-K119R* (LV252) *cdk9-T212E htb1-K119R* (LV256), and *mcs6-S165A htb1-K119R* (LV254) cells. (D) Quantification of abnormal septation patterns in strains of indicated genotypes (JTB62-1, JTB67-1, JTB325, JTB326, JTB331, JTB335, JTB377, JTB333 respectively). Error bars represent standard deviations from 2 independent experiments; at least 200 cells were counted in each. (E) Flocculation of indicated *htb1-FLAG* strains (JTB62-1, JTB67-1, JTB325, JTB326) was quantified as described in Materials and Methods. Error bars denote standard deviations from 2 independent experiments.

Biochemical analyses revealed a reciprocal relationship between Spt5-P and H2Bub1, suggestive of a positive feedback loop. The synthetic phenotype of *spt5-T1A set1Δ* double mutants, moreover, indicated that the Cdk9-Spt5 axis was one of two partially redundant gene-regulatory pathways downstream of H2Bub1. To detect and characterize direct genetic interactions between P-TEFb and H2Bub1, we tested sensitivity of a *cdk9^as^ htb1-K119R* double mutant strain to 3-MB-PP1. Unexpectedly, cells bearing a *cdk9^as^* allele were *less* sensitive to growth inhibition by 3-MB-PP1 in an *htb1-K119R*, compared to an *htb1^+^*, background (Figure 5A). In contrast, the *htb1-K119R* mutation exacerbated 3-MB-PP1-sensitivity of *mcs6^as^* and *lsk1^as^* cells (data not shown). Therefore, lack of H2Bub1 specifically reduced dependency on active Cdk9, consistent with the two pathways acting antagonistically. Absence of H2Bub1 did not fully bypass the requirement for Cdk9 activity, however, because higher doses of 3-MB-PP1 were still capable of arresting proliferation of *cdk9^as^ htb1-K119R* cells.

In the absence of inhibitory analogs, *cdk9^as^ htb1-K119R* cells displayed hyperseptated and branched morphologies characteristic of H2Bub1-defective mutants. After treatment with 10 µM 3-MB-PP1 for 7 hr, however, cell morphology reverted to normal (Figure 5B). This effect was reversible; after washout of 3-MB-PP1 and return to drug-free medium, the “reverted” *cdk9^as^ htb1-K119R* cells re-acquired an aberrant, hyperseptated morphology.

To confirm that impairment of Cdk9 function could suppress phenotypes caused by the absence of H2Bub1, we combined a *cdk9-T212A* mutation, which prevents activation of Cdk9 by a CAK but allows cell viability [50], with *htb1-K119R* or *brl2Δ*. As was the case with chemical inhibition of Cdk9, *cdk9-T212A* rescued morphologic phenotypes of H2Bub1-defective mutants (Figure 5C, 5D). Importantly, there was no suppression of *htb1-K119R* or *brl2Δ* by *lsk1* loss-of-function alleles (Figure 5D and data not shown), establishing the specificity of the interaction between Cdk9 and H2Bub1. Similarly, neither *cdk9-T212E* nor *mcs6-S165A* could correct the aberrant morphologies of *htb1-K119R* cells (Figure 5C). Reversal of septation phenotypes by *cdk9-T212A* was accompanied by loss of the flocculation observed in *htb1-K119R* single mutants; ∼15% of wild-type or *cdk9-T212A htb1-K119R* double mutant cells settled out of liquid cultures after 1 hr, compared to ∼60% of *cdk9^+^ htb1-K119R* cells (Figure 5E), consistent with suppression of *htb1-K119R* by reduction of Cdk9 activity.

**Figure 6:**
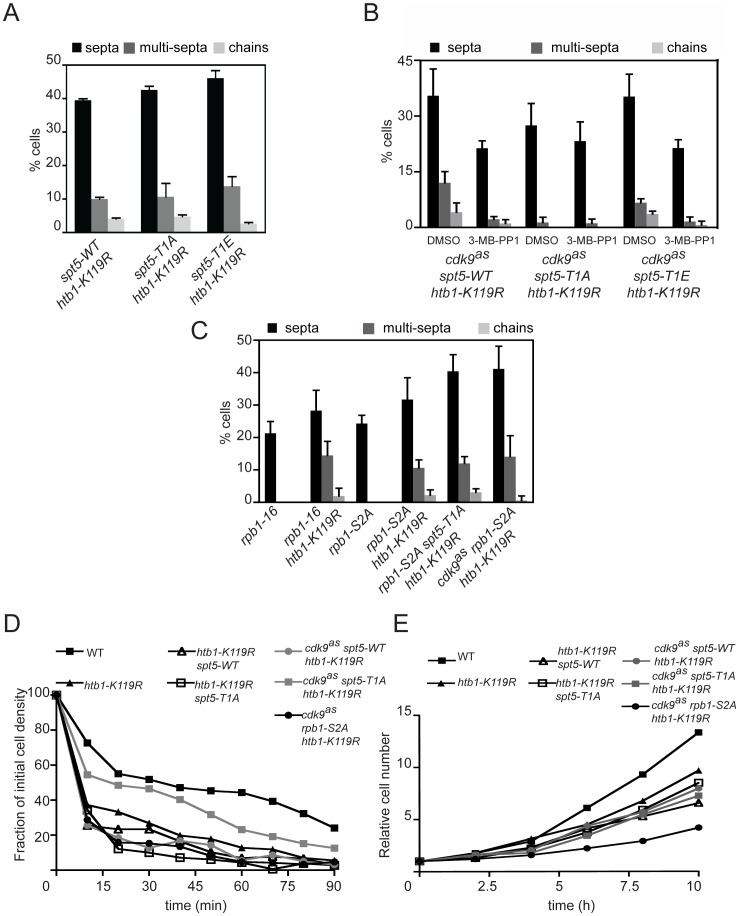
Cdk9 activity towards multiple substrates is required for abnormal morphologies of H2Bub1-deficient cells. (A) Quantification of abnormal septation patterns in the indicated *htb1-FLAG* strains (JTB378, JTB351, JTB379, JTB353, JTB380, JTB355). Error bars represent standard deviations from 2 independent experiments; at least 200 cells were counted in each. (B) As in (A) for the indicated *cdk9^as^* strains (KL289, KL291, KL293). Cells were grown in the presence of either DMSO or 3-MB-PP1 as described in Figure 5B. (C) As in (A) for the indicated *htb1-FLAG* strains (MS260, MS256, MS261, MS257, MS272, MS259). (D) Flocculation of indicated strains (JS78, JTB67-1, JTB351, JTB353, KL291, KL293, MS259) was quantified as described in Materials and Methods. (E) Growth rates in liquid rich medium (YES) were measured for strains analyzed in (D).

We next asked if aberrant morphologies due to *htb1-K119R* could be suppressed by mutations in the known Cdk9 substrates: Spt5 and Rpb1 [51]. By itself, *spt5-T1A* did not modify *htb1-K119R* phenotypes (Figure 6A), implicating another Cdk9 target (or targets) in the observed suppression. We attempted to verify this by combining *cdk9^as^* with *spt5-T1A* in an *htb1-K119R* background, to allow selective inhibition of Cdk9 in the absence of Spt5-Thr1 phosphorylation. Unexpectedly, the triple-mutant cells grown in the *absence* of inhibitory analogs had nearly normal morphology—a septation index close to that of the wild-type strain, <5% multiseptated cells and no chained cells (Figure 6B). In vitro, the activity of Cdk9^as^ was reduced ∼3-fold relative to that of wild-type Cdk9, even in the absence of drugs (Figure S10). This is likely to be due to decreased affinity for ATP—a known consequence of the gatekeeper mutation in other AS kinases [72], [73]. The data suggest that loss of Spt5-Thr1 phosphorylation, combined with the partial reduction of Cdk9 activity, suppressed morphological abnormalities caused by loss of H2Bub1.

These results reveal multiple, distinct roles for Spt5-Thr1 phosphorylation in mediating regulatory interactions between H2Bub1 and Cdk9. When Cdk9 activity levels are normal, Spt5-P serves, in combination with Set1, to promote H2Bub1 functions in maintaining normal cell morphology (Figure 3C, 3D). When H2Bub1 is absent, however, Spt5-P, together with at least one other Cdk9-dependent pathway, contributes to aberrant and excessive septation (Figure 6B). A similar distinction between specific elimination of Spt5-P and a general reduction in Cdk9 activity is apparent in a *set1Δ* background, in which *spt5-T1A* resulted in cell-morphology defects but *cdk9-T212A* had no effect (Figure 3D). Thus, the positive feedback between H2Bub1 and Cdk9, involving a single Cdk9 target (Spt5), is genetically distinguishable from the antagonism, which involves multiple targets (Spt5 and one or more others).

Cdk9 also contributes to phosphorylation of Ser2 and Ser5 of RNAPII CTD repeats [50], [51], [74]. An *rpb1-S2A* mutation that replaced Ser2 in all repeats with Ala in the context of a truncated but still functional CTD [53] failed to suppress *htb1-K119R* in either a *cdk9^+^* or *cdk9^as^* background (Figure 6C). Combined loss of both Spt5-Thr1 and Rpb1-Ser2 in a *cdk9^+^* background also did not suppress *htb1-K119R* septation phenotypes. Similarly, in a *cdk9^as^* background, *spt5-T1A* but not *rpb1-S2A* suppressed flocculation due to an *htb1-K119R* mutation (Figure 6D). Therefore, diminished Cdk9 activity must rescue phenotypes caused by H2Bub1 loss through reduced phosphorylation of Spt5-Thr1 *and* another target (or targets) besides Rpb1-Ser2.

The suppression of hyperseptation and flocculation was not simply a consequence of reduced growth rate, because hyperseptated, flocculating *cdk9^+^ spt5-T1A htb1-K119R* cells and suppressed *cdk9^as^ spt5-T1A htb1-K119R* cells grew at similar rates in liquid culture. Moreover, the non-suppressed, *cdk9^as^ rpb1-S2A htb1-K119R* strain grew more slowly than did the suppressed strain (Figure 6E). These data indicate that suppression of *htb1-K119R* is a specific consequence of reduced Cdk9 activity.

### RNAPII redistribution due to *htb1-K119R* depends on Cdk9 activity

**Figure 7:**
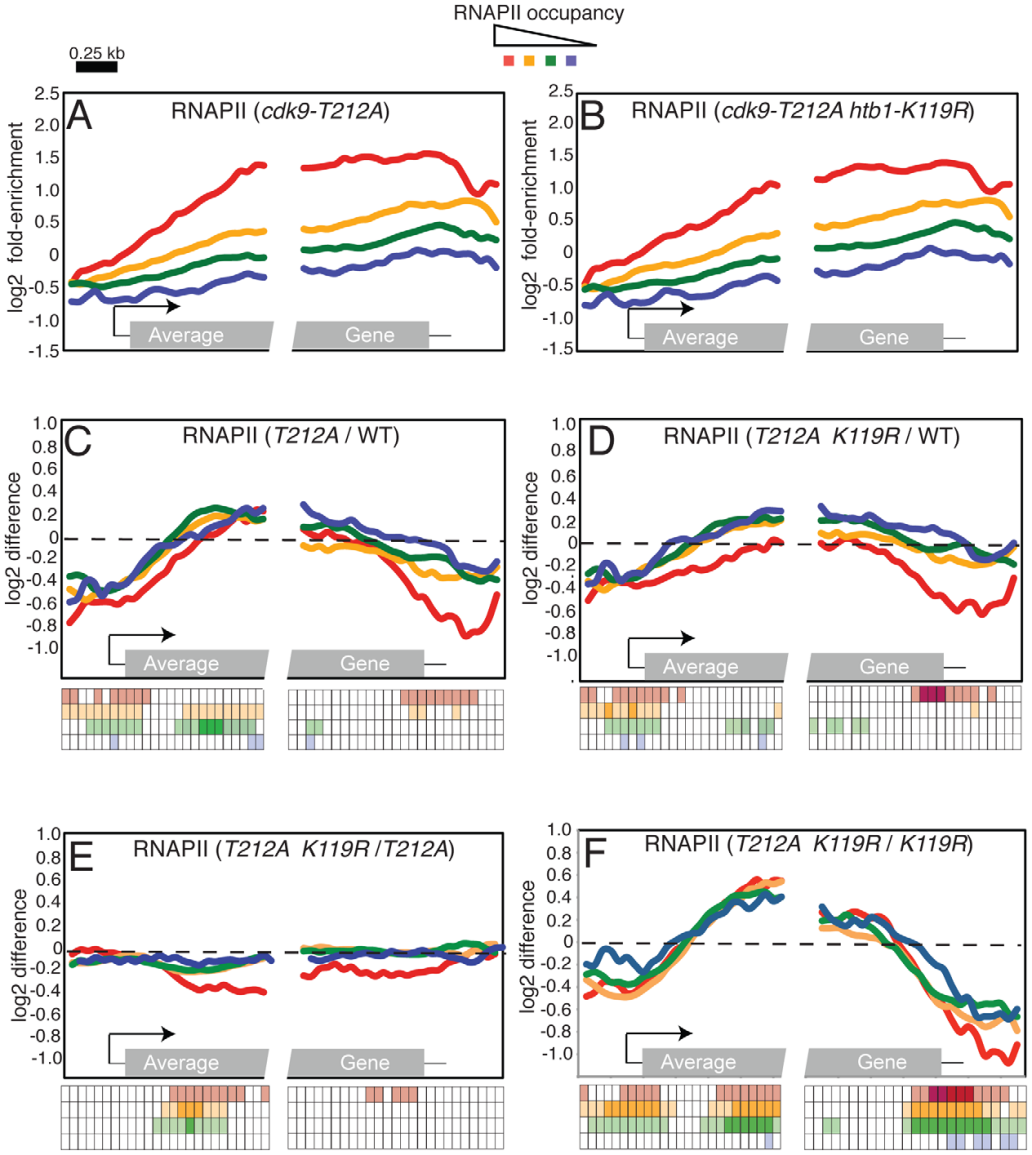
Opposing effects of H2Bub1 and Cdk9 activity on RNAPII distribution revealed by ChIP–chip. (A) Average distribution of RNAPII at 540 *S. pombe* genes, as determined by ChIP-chip in a *cdk9-T212A* strain (JTB325). Genes were grouped according to total levels of RNAPII enrichment. (B) As in (A) for *cdk9-T212A htb1-K119R* (JTB326). (C) Average distributions of differences between *cdk9-T212A* (JTB325) and wild-type (JTB62-1) RNAPII enrichment grouped according to RNAPII enrichment in wild-type cells. (D) As in (C) for differences between *cdk9-T212A htb1-K119R* and wild-type RNAPII enrichment. (E) As in (C) for differences between *cdk9-T212A htb1-K119R* and *cdk9-T212A* RNAPII enrichment. (F) As in (C) for differences between *cdk9-T212A htb1-K119R* and *htb1-K119R* RNAPII enrichment. The keys below C-F illustrate the statistical significance of the differences for each group at 50 positions along the average gene. The rows of the key are color-coded according to the graph. Open squares denote p>0.01; light shading denotes 0.01>p>10exp-5; dark shading denotes p<10exp-5 (one-sample t-tests; μ_0_ = 0). Note that there is only light shading for the last row (corresponding to the blue curve).

**Figure 8:**
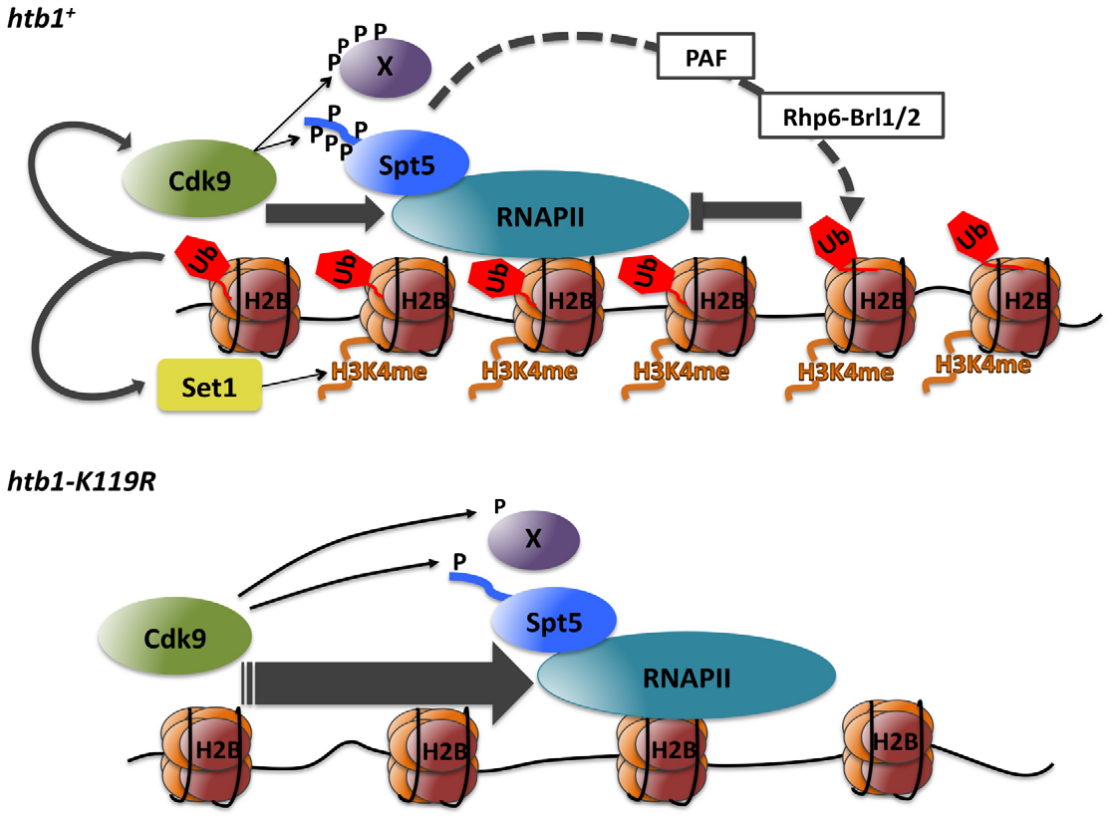
Model depicting positive and negative interactions between P-TEFb and H2Bub1 during transcript elongation (see text for details).

Our results thus far indicated that loss of H2Bub1 partially alleviated the cellular requirement for Cdk9 activity and that, conversely, selective impairment of Cdk9 function suppressed phenotypes of H2Bub1-defective mutants. This mutual suppression led us to hypothesize that P-TEFb and H2Bub1 might act antagonistically to control transcript elongation. We tested such an interaction by asking whether a reduction in Cdk9 activity could reverse the altered RNAPII distribution observed in an *htb1-K119R* mutant. We analyzed genome-wide RNAPII crosslinking by ChIP-chip in *cdk9-T212A* and *cdk9-T212A htb1-K119R* strains (Figure 7; Datasets S4 and S5). The *cdk9-T212A* mutation caused a global redistribution of RNAPII occupancy that was essentially opposite to that observed in the *htb1-K119R* strain: an increase in RNAPII density in the 5′ halves of genes, and a decrease toward the 3′ ends of genes that was particularly significant at highly transcribed loci (compare Figure 7C and Figure 1D). Impaired Cdk9 activity also led to reversal of the RNAPII 3′ shift caused by *htb1-K119R* (Figure 7F). Global RNAPII occupancy profiles in *cdk9-T212A htb1^+^* and *cdk9-T212A htb1-K119R* mutants were nearly identical, suggesting that, when Cdk9 activity was reduced, H2Bub1 had little or no effect on RNAPII dynamics at the majority of genes (Figure 7A–7E). Loss of H2Bub1 reduced RNAPII occupancy within the 5′ halves of genes even in the context of reduced Cdk9 activity, however, suggesting that this effect is partially Cdk9-independent (Figure 7D, 7E). Nonetheless, full Cdk9 activity is required for the increase in RNAPII density at the 3′ ends of genes in the absence of H2Bub1 (Figure 7E). These data argue that P-TEFb and H2Bub1 antagonize one another to govern the distribution of elongating RNAPII within genes (Figure 8).

## Discussion

Here we combined chemical genetics and genomics to reveal reciprocal relationships between a CDK and a co-transcriptional histone modification in fission yeast. First, we showed interdependence of Cdk9-mediated Spt5 phosphorylation and H2Bub1—evidence for co-regulation governed by positive feedback. Importantly, we found that this feedback loop and the histone methyltransferase Set1 support independent functions of H2Bub1 in vivo. Second, we uncovered genetic interactions between mutations that compromise either Cdk9 activity or H2Bub1, which indicate opposing functions. To explain this paradox we propose a homeostatic mechanism that ensures optimal balance of P-TEFb and H2Bub1 functions during elongation.

### A positive feedback loop linking Spt5-P and H2Bub1

A key Cdk9 substrate in the H2Bub1 pathway is Spt5, an ancient component of the transcription machinery that promotes RNAP processivity [75]–[77]. Eukaryotic Spt5 orthologs contain carboxyl-terminal sites phosphorylated by P-TEFb; *S. pombe* Spt5 has a contiguous array of nonapeptide repeats, which are phosphorylated at Thr1 by Cdk9 in vitro [52]. We showed that Cdk9 activity is necessary for phosphorylation of Spt5-Thr1 in vivo, consistent with genetic epistasis between Spt5-CTD truncation and selective Cdk9 inhibition [51]. Furthermore, reduction of Cdk9 activity or mutation of Spt5-Thr1 to Ala reduced H2Bub1. In budding yeast, Spt5 phosphorylation promotes H2Bub1 through recruitment of the PAF complex [37], [38], and Spt5 also influences H2Bub1 in mammalian cells [31], [78], suggesting a conserved signaling pathway.

Cdk9-dependent phosphorylation of the Rpb1 CTD has been implicated in formation of H2Bub1 in mammalian cells [30], [79]. The sites phosphorylated by Cdk9 within the CTD repeat—Ser2 and Ser5—are essential for cell viability, however, precluding a definitive proof of their involvement in this pathway. Because Ser2 is not essential in *S. pombe* [53], [68], we have been able to test its contribution to H2Bub1 more directly; the *rpb1-S2A* mutation had no effect on H2Bub1 levels (data not shown). Understanding the relative contributions of the Rpb1 CTD and other Cdk9 substrates in directing H2Bub1 will probably require better tools for study of metazoan P-TEFb and its targets in vivo.

The identification of Spt5-Thr1 phosphorylation as a specific and sensitive indicator of Cdk9 activity allowed us to uncover another, unexpected relationship between P-TEFb and H2Bub1 in vivo: Cdk9-mediated phosphorylation of Spt5 depends on H2Bub1. ChIP analysis suggested a possible explanation—decreased recruitment of Cdk9 to H2Bub1-deficient chromatin. In mammalian cells, basal association of P-TEFb with the HIV-1 promoter decreased upon depletion of the Bre1 ortholog RNF20, consistent with a conserved role for H2Bub1 in P-TEFb recruitment [80]. Based on our data and results in budding yeast, we propose that a positive feedback loop connects Cdk9 activity and H2Bub1 through the sequence: 1) phosphorylation of Spt5 by Cdk9; 2) PAF recruitment by Spt5-P; 3) recruitment and/or activation of the Rhp6 (*S. pombe* ortholog of Rad6)-Brl1/2 complex by PAF, to generate H2Bub1; and 4) Cdk9 recruitment, leading to reiteration of the cycle (Figure 8). Although the mechanism by which H2Bub1 facilitates Cdk9 recruitment remains to be determined, there is precedent in metazoans for P-TEFb association with modified histones [44], [45].

### Two independent protein modification pathways downstream of H2Bub1

Our results indicate that H3K4me and Spt5-P are independently stimulated by H2Bub1 in vivo. Importantly, elimination of either modification alone did not cause septation phenotypes, but their combined ablation phenocopied the aberrant cell morphologies produced by H2Bub1 loss. Therefore, we have identified a molecular intermediate—Spt5-P—in the H3K4me-independent pathway downstream of H2Bub1 (Figure 8). A challenge for the future will be to ascertain how Set1 cooperates with Cdk9, acting through Spt5-Thr1, to regulate gene expression.

### Cdk9-H2Bub1 antagonism during RNAPII elongation

The mutually reinforcing relationship between Cdk9 activity and H2Bub1 seemed to predict the two would act in concert during transcription. Instead, genetic analysis indicated they work in opposition: loss of H2Bub1 partially relieved the requirement for Cdk9 activity in vivo, and reduction in Cdk9 activity suppressed cell-morphology phenotypes of H2Bub1-deficient mutants. Simultaneous mutation of two known sites of Cdk9-dependent phosphorylation (Spt5-Thr1 and Rpb1-Ser2) did not suppress *htb1-K119R*, implicating at least one other Cdk9 target in opposing H2Bub1 function. Consistent with the genetic interaction, Cdk9 and H2Bub1 exerted inverse effects on global RNAPII distribution: whereas RNAPII density shifted toward the 3′ ends of genes in an *htb1-K119R* mutant, lowering Cdk9 activity produced the opposite effect in an *htb1^+^* background, and prevented the 3′ shift in the double-mutant strain.

The antagonism we observe between Cdk9 activity and H2Bub1 is incomplete—loss of H2Bub1 did not entirely bypass the essential function of Cdk9, and led to altered RNAPII occupancy in the 5′ portions of genes even when Cdk9 activity was reduced. Conversely, although the presence of *cdk9-T212A* eliminated aberrant cell morphologies associated with *htb1-K119R*, the growth rate of double mutant cells was slower than that of either single mutant (data not shown). These results imply that other pathways can modulate the functions of Cdk9 and H2Bub1 in transcript elongation.

Our results strengthen the notion that P-TEFb activity is required to overcome repressive effects of histone modifications, first suggested by work in budding yeast. In *S. cerevisiae*, the instructive genetic interactions occurred between *BUR1* and the H3K36 methyltransferase Set2; lethality of a *bur1* null mutation was suppressed by deletion of *SET2* [34], [48]. In fission yeast, however, *set2* deletion did not suppress growth arrest due to inhibition of Cdk9^as^ (L.V. and R.P.F., unpublished observations). Interestingly, *bur1Δ* was also weakly suppressed by deletion of *RAD6* [48], implying that a regulatory interaction between P-TEFb and H2Bub1 might be conserved in budding yeast.

In mammalian cells, the Bre1/Brl2 ortholog RNF20 selectively represses transcription of genes implicated in tumorigenesis; its knockdown reduces H2Bub1, enhances the transcriptional response to growth factors and promotes cellular transformation [13]. These effects were recently shown to depend on the transcription elongation factor TFIIS [81]. This extends the parallel between mammals and fission yeast, by suggesting that H2Bub1 selectively modulates gene expression in both settings by restraining transcript elongation, albeit through different factors—TFIIS or P-TEFb, respectively. Given the established dependence of H2Bub1 on CDK9 activity in mammalian cells [30], [31], it will now be important to ask if metazoan P-TEFb function is also reciprocally influenced by H2Bub1.

### Evidence for a chromatin-based checkpoint in RNAPII elongation

How can we reconcile biochemical evidence for mutual dependence of Cdk9 activity and H2Bub1 with genetic evidence for their antagonism? We propose a homeostatic mechanism whereby Cdk9-H2Bub1 interdependence ensures a balance between their opposing functions. In this scenario, Cdk9 promotes H2Bub1 to regulate its own effects on elongation (Figure 8). In an *htb1-K119R* mutant, the balance is disrupted, resulting in Cdk9-dependent accumulation of RNAPII in 3′ regions of genes. We suggest this pattern reflects unchecked elongation that is poorly responsive to mRNA processing signals, and may imply enhancement of the RNAPII pausing normally associated with mRNA 3′ end processing [82], [83]. H2Bub1 has been linked to various aspects of RNA processing in budding yeast and in mammalian cells, including splicing, 3′ end formation, and nuclear export [30], [57], [84]–[86]. In fission yeast, Cdk9 has been implicated in mRNA 5′-end formation, through its role in recruiting a capping enzyme to transcribed chromatin [87]. Still to be determined are the full range of H2Bub1-dependent functions that depend on P-TEFb activity and the mechanism(s) by which H2Bub1 and Cdk9 influence each other.

In cell division, faithful genome duplication and segregation are ensured by checkpoints—extrinsic signaling pathways that enforce dependency between intrinsically independent events [88]. Transcription and mRNA-processing could be intrinsically coupled through chromatin structure, because removal of nucleosomal barriers is likely to be a limiting step for elongation [89]. Here we have uncovered a role for a covalent chromatin modification, H2Bub1, in regulating elongation through an extrinsic pathway involving Cdk9 and Spt5. We propose a checkpoint-like function of H2Bub1, to set thresholds of Cdk9 activity and Spt5-P required for elongation and mRNA maturation, with the balance between opposing functions of Cdk9 and H2Bub1 maintained by virtue of their interdependence. The absence of H2Bub1 (and consequent decreases in Spt5-P and H3K4me) might hinder mRNA processing events normally coupled to Cdk9-mediated phosphorylations, without fully alleviating the Cdk9-dependence of elongation; “rescue” of these defects by reducing Cdk9 activity would be analogous to rescue of cell-cycle checkpoint mutants by drugs or mutations that slow another process.

## Materials and Methods

### *S. pombe* strains and media

Strains used in this study are listed in Table S2. Cells were grown in YE medium containing 250 mg/L each of adenine, leucine, histidine, and uracil (YES). Strains were constructed by standard genetic techniques [90].

### Immunological methods

To detect Spt5 phosphorylation by immunoblotting, *S. pombe* whole-cell extracts were prepared as previously described [37], [91]. To detect chromatin modifications, cells were lysed in trichloroacetic acid, as described [92]. The Spt5-P antibody was raised against the peptide acetyl-NSGNK[pT]PAWNVGNK[pT]PAWNSC-amide injected into rabbits, and purified from whole serum after depletion with the same peptide in unphosphorylated form immobilized on resin, by adsorption to and elution from resin-bound acetyl-AWNSGSK[pT]PAWNSGSC-amide by 21st Century Biochemicals (Marlboro, MA). Other antibodies are listed in Text S1.

### Chromatin immunoprecipitation and microarray analysis

ChIP was carried out as described previously [12], [93]. Amplification, labeling, and hybridization of control and immunoprecipitated DNA samples for ChIP-chip were carried out as described previously and normalized data are included as Datasets S1, S2, S3, S4, S5 [94]. Further details are included in Text S1. Sequences of primers used for qPCR analysis are available upon request.

### Microscopy

*S. pombe* cells were fixed and stained with diamino-phenylindole (DAPI) and calcofluor as described previously [51]. Cells were viewed using a Leica DM5000b microscope and photographed with a CCD camera. Images were processed using Volocity software.

### Chemical genetic methods

Cultures were treated with 3-MB-PP1 at indicated doses, or with vehicle (DMSO), for indicated times at 30°C prior to extract preparation. Dose-response to 3-MB-PP1 was determined as previously described [51]. To analyze effects of 3-MB-PP1 on cell morphologies, cells were grown at 30°C to OD_600_∼0.1 in YES and treated with 10 µM 3-MB-PP1 or DMSO for 7 hr, then fixed and stained for microscopy immediately or after return to growth in drug-free YES for 7 hr.

### Flocculation assays

Assays were carried out as described previously [95].

## Supporting Information

Dataset S1 Microarray data for ChIP-chip performed with a H2Bub1 antibody (wild-type) and a RNAPII antibody (wild-type and *htb1-K119R*).
(ZIP)

Dataset S2 Microarray data for ChIP-chip performed with a histone H3 antibody (wild-type).
(ZIP)

Dataset S3 Microarray data for ChIP-chip performed with a histone H3 antibody (*htb1-K119R*).
(ZIP)

Dataset S4 Microarray data for ChIP-chip performed with a RNAPII antibody (*cdk9-T212A*).
(ZIP)

Dataset S5 BED file containing microarray data for ChIP-chip performed with a RNAPII antibody (*cdk9-T212A htb1-K119R*).
(ZIP)

Figure S1 Specificity of H2Bub1 antibody for ChIP in *S. pombe*. (A) H2Bub1 occupancy was measured by ChIP in wild-type (JTB62-1) and *htb1-K119R* (JTB67-1) strains and quantified at the inactive *hsp16* ^+^ gene and constitutive *act1* ^+^ gene. Values were normalized to H2B-FLAG occupancy measured in parallel to control for differences in nucleosome density. Positions of primer pairs used for qPCR are indicated in schematic at top. (B) Immunoblots of whole-cell extracts from untagged (JTB204), *htb1-FLAG* (JTB62-1), and *htb1-K119R-FLAG* (JTB67-1) strains using the indicated antibodies.
(PDF)

Figure S2 Loss of H2Bub1 does not significantly alter nucleosome density in *S. pombe*. (A) Average distribution of histone H3 at 540 *S. pombe* genes, as determined by ChIP-chip in a wild-type strain (JTB62-1). Genes were grouped according to total levels of RNAPII enrichment (see key at top). The grey box in the “average gene” representation at bottom denotes the gene coding region; 5′ and 3′ untranslated regions are denoted by thin black lines. The arrow denotes the transcription start site. (B) As in (A), determined in an *htb1-K119R* mutant strain (JTB67-1). Gene groupings were created using wild-type RNAPII enrichment values. (C) Average distributions of differences between mutant and wild-type histone H3 enrichment grouped according to RNAPII enrichment in wild-type cells. The key below the graph illustrates the statistical significance of the differences for each group at 50 positions along the average gene. The rows of the key are color-coded according to the graph. Open squares denote p>0.01; light shading denotes 0.01>p>10exp-5 (one-sample t-tests; μ_0_ = 0).
(PDF)

Figure S3 Chromatin association of Rhp6 and the PAF protein Rtf1 requires Cdk9 activity. (A) Rhp6-TAP occupancy was measured by ChIP in the indicated strains and quantified at the *eng1^+^* gene by qPCR. Enrichment is plotted as percentage of the input signal for each primer pair. Positions of PCR primer pairs are indicated in the schematic at top. (B) Rtf1-TAP occupancy was measured by ChIP in the indicated strains and quantified at the *act1^+^* gene by qPCR. Enrichment is plotted as percentage of the input signal for each primer pair. Positions of PCR primer pairs are indicated in the schematic at top. In all cases, treatment of the *cdk9^as^* strains was with either DMSO (-) or 20 µM 3-MB-PP1 for 3 hr prior to harvesting. Error bars denote standard deviations from three independent experiments.
(PDF)

Figure S4 Validation of Spt5 as an exclusive Cdk9 target using a phospho-specific Spt5 antibody. (A) Kinase reactions containing recombinant GST-Spt5 substrate and indicated combinations of purified, recombinant Cdk9/Pch1 and Csk1 were analyzed by immunoblotting with: affinity-purified antibody specific for phosphorylated Spt5 (Spt5-P); crude serum specific for wild-type Spt5 CTD repeats, regardless of phosphorylation state (Spt5-CTD; see Text S1); or anti-GST antibody (GST). (B,C) Immunoblots of whole-cell extracts from indicated *spt5-myc* strains after treatment with increasing concentrations of 3-MB-PP1. Antibodies are indicated at right. (D) Both phospho-isoform- and pan-specific total Spt5 antibodies are specific for the wild-type nonapeptide repeat sequence of the Spt5 CTD. Whole-cell extracts from the indicated strains were probed with affinity-purified antibody (top, “Spt5-P”) or crude serum (bottom, “Spt5”) from a rabbit immunized with an Spt5-CTD phosphopeptide (see Materials and Methods). Spt5 antibodies discriminate against nonapeptides with substitutions at the Thr1 position, but not against unphosphorylated, wild-type Spt5-CTD (see Figure 3A), illustrating why phospho-site mutant proteins cannot be used as controls for specificity of phosphospecific antibodies.
(PDF)

Figure S5 The FACT subunit Pob3 has functions distinct from those of H2Bub1. (A) Immunoblots of whole-cell extracts from wild-type (JTB204), *brl2Δ* (JTB331), *cdk9-T212A* (HD7-24), and *pob3Δ* (JTB281-1) strains using the indicated antibodies. (B) Quantification of abnormal septation in strains of indicated genotypes (JTB317 and JTB318, respectively). Error bars represent standard deviations from 2 independent experiments; at least 200 cells were counted in each.
(PDF)

Figure S6 Anti-Spt5-P specifically immunoprecipitates Spt5-P from wild-type and *spt5-myc* strains. (A) Spt5-P immunoprecipitation from whole-cell extracts. Extracts from indicated strains (bottom) were incubated with or without anti-Spt5-P (top), and immunoprecipitates were analyzed by immunoblotting with antibodies indicated at right. The *cdk9^as^* strain was treated prior to extract preparation with either DMSO or 5 µM 3-MB-PP1, as indicated at bottom, for 20 min. For each treatment we analyzed 10% of input extract (“input”), 10% of supernatant after precipitation (“sup”) and 50% of immunoprecipitated material (“IP”). Antibodies are indicated at right. (B) ChIP of Spt5-P and Spt5-myc at *eng1* ^+^. Patterns of Spt5-P occupancy on *eng1^+^* are similar in wild-type (“WT,” top; JS78) and *spt5-myc* (middle; CS111) strains. Distribution of total Spt5 on *eng1^+^* was measured in the *spt5-myc* strain (bottom). (C) Spt5-myc (top) and Spt5-P (middle) occupancies were measured by ChIP in a *cdk9^as^ spt5-myc* strain and quantified at indicated positions within *eng1* ^+^ by qPCR. Cells were treated prior to lysis with either DMSO or 5 µM 3-MB-PP1 for 20 min. Enrichment is plotted as percentage of input signal for each primer pair. The ratios of Spt5-P/Spt-myc at each position are plotted at bottom.
(PDF)

Figure S7 Impact of H2Bub1 on Spt5 phosphorylation and Cdk9 recruitment at *nup189^+^* and *SPB354.10^+^*. (A,B) Spt5 phosphorylation was measured by ChIP using anti-Spt5-P in *spt5-myc* (MS265; black bars) and *spt5-myc htb1-K119R* (LV239; gray bars) strains and quantified at the indicated genes by qPCR. In all graphs of this figure the enrichment is plotted as percentage of the input signal for each primer pair, positions of PCR primer pairs within coding regions are indicated in the schematic at top, error bars denote standard deviations from 3 independent experiments and asterisks denote a significant difference between wild-type and mutant (“*” p<0.04, “**” p<0.02; unpaired t-test). (C,D) Spt5-myc occupancy was measured by ChIP as in C and D. (E,F) Spt5-P enrichment normalized to total Spt5-myc occupancy. (G,H) Cdk9-myc occupancy was measured by ChIP in *cdk9-myc* (MS264; black bars) and *cdk9-myc htb1-K119R* (KL259; gray bars) strains.
(PDF)

Figure S8 ChIP of Spt5-P, Spt5-myc, and Cdk9-myc at an intergenic sequence. (A) Occupancy of total Spt5, phospho-Spt5 and Cdk9 was measured by ChIP using anti-myc or anti-Spt5-P in wild-type strains (MS265 for Spt5-myc and Spt5-P IP; MS264 for Cdk9-myc IP), followed by qPCR using primers covering the 5′ end of *eng1* (*eng1-1*) and an intergenic region as control (*control*). In all graphs of this figure the enrichment is plotted as percentage of the input signal for each primer pair and error bars denote standard deviations from 3 independent experiments. (B) Same experiment as in (A) in *htb1-K119R* strains (LV239 for Spt5-myc and Spt5-P IP; KL259 for Cdk9-myc IP).
(PDF)

Figure S9 Impact of H2Bub1 on RNAPII occupancy and mRNA expression at individual genes. (A-D) RNAPII occupancy was measured by ChIP in wild-type (JTB62-1; black bars) and *htb1-K119R* (JTB67-1; gray bars) strains and quantified at the indicated genes by qPCR. Enrichment is plotted as percentage of the input signal for each primer pair, and positions of PCR primer pairs within coding regions are indicated in the schematic at top of each graph. (E) Levels of mRNA from the indicated genes were quantified by qRT-PCR and normalized to *act1^+^*. Throughout this figure error bars denote standard deviations from 3 independent experiments and asterisks denote a significant difference between wild-type and mutant (“*” p<0.04, “**” p<0.02; unpaired t-test).
(PDF)

Figure S10 Kinase activity of Cdk9 and Cdk9^as^ complexes purified from *S. pombe*. Lysates from *pcm1-myc* (CS145) or *cdk9^as^ pcm1-myc* (CS165) strains were incubated either with anti-myc-bound protein G beads (α-myc-beads) to co-immunoprecipitate Cdk9 bound to Pcm1-myc or with protein G alone (mock). Input lysate amounts in each immunoprecipitation were varied as indicated. Immunoprecipitates were analyzed by immunoblotting (top panels) and for Rpb1-CTD kinase activity (middle panels). Phosphorylation signals were visualized by autoradiography and quantified with a phosphorimager (bottom).
(PDF)

Table S1 RNAPII distributions at H2Bub1-stimulated genes in the *htb1-K119R* strain.
(DOC)

Table S2 *S. pombe* strains used in this study.
(DOC)

Text S1 Supplemental materials and methods.
(DOC)
