# Supplementary figures and images for: A Positive Feedback Loop Links Opposing Functions of P-TEFb/Cdk9 and Histone H2B Ubiquitylation to Regulate Transcript Elongation in Fission Yeast
Source: PLoS Genet. 2012 Aug 2;8(8):e1002822. doi: 10.1371/journal.pgen.1002822 (PMC3410854; doi:10.1371/journal.pgen.1002822)

A

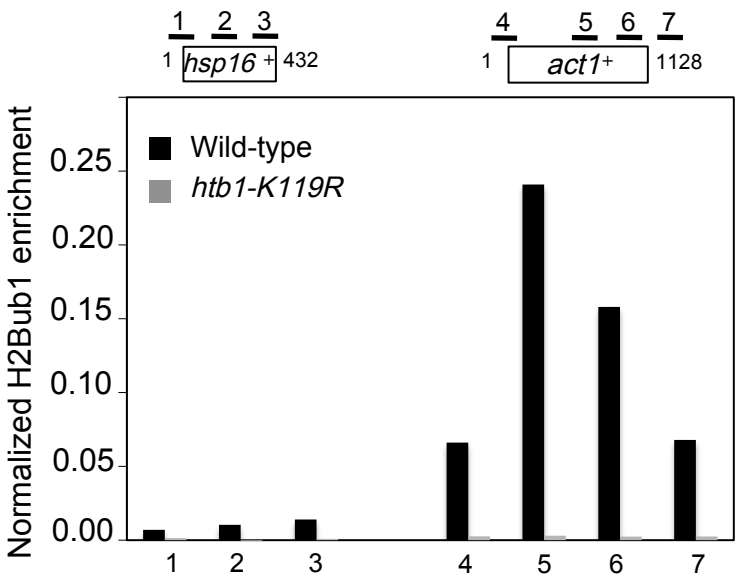

B

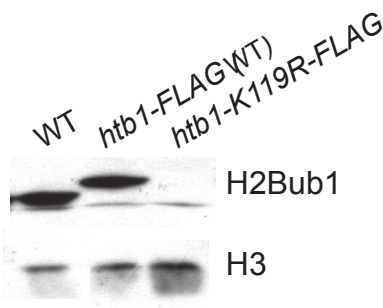

Supplement: Figure S1 — Specificity of H2Bub1 antibody for ChIP in S. pombe. (A) H2Bub1 occupancy was measured by ChIP in wild-type (JTB62-1) and htb1-K119R (JTB67-1) strains and quantified at the inactive hsp16 + gene and constitutive act1 + gene. Values were normalized to H2B-FLAG occupancy measured in parallel to control for differences in nucleosome density. Positions of primer pairs used for qPCR are indicated in schematic at top. (B) Immunoblots of whole-cell extracts from untagged (JTB204), htb1-FLAG (JTB62-1), and htb1-K119R-FLAG (JTB67-1) strains using the indicated antibodies. (PDF) [file pgen.1002822.s006.pdf]

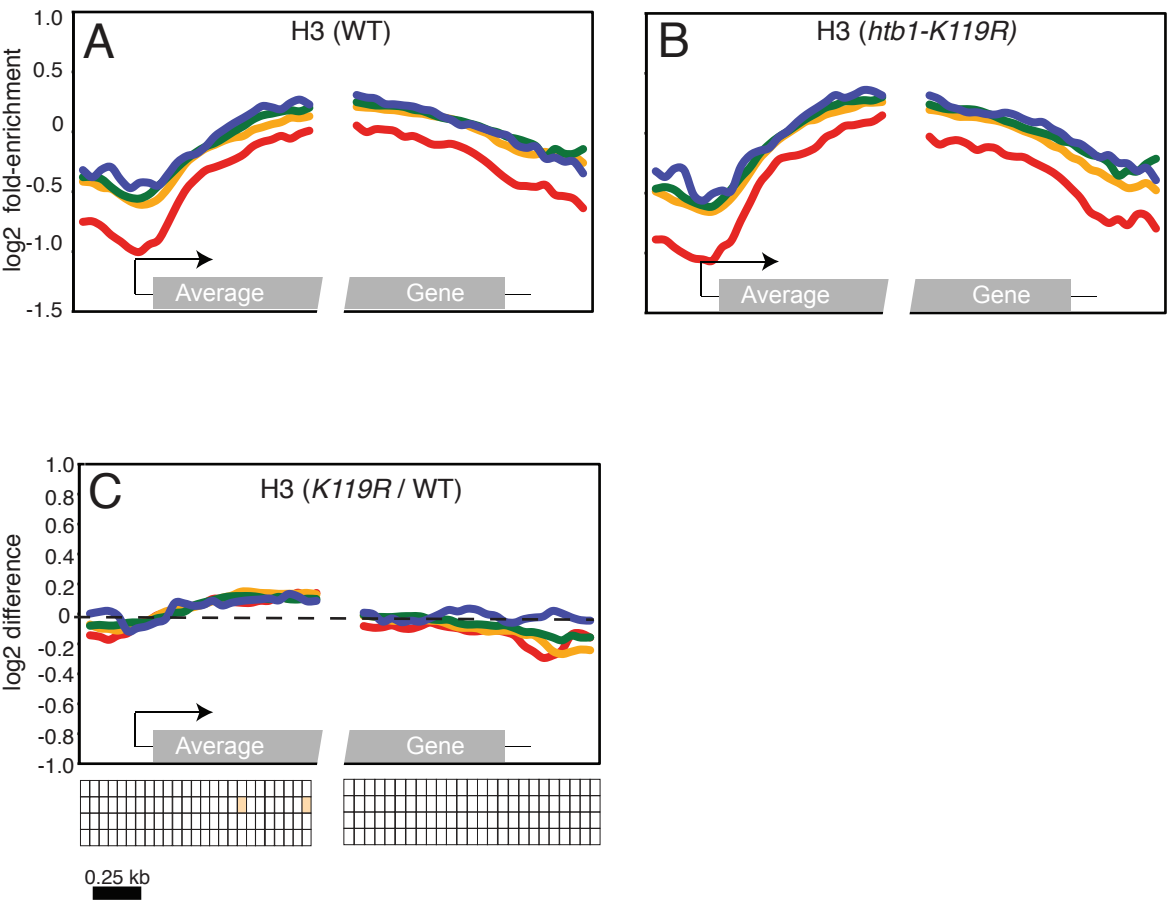

Supplement: Figure S2 — Loss of H2Bub1 does not significantly alter nucleosome density in S. pombe. (A) Average distribution of histone H3 at 540 S. pombe genes, as determined by ChIP-chip in a wild-type strain (JTB62-1). Genes were grouped according to total levels of RNAPII enrichment (see key at top). The grey box in the “average gene” representation at bottom denotes the gene coding region; 5′ and 3′ untranslated regions are denoted by thin black lines. The arrow denotes the transcription start site. (B) As in (A), determined in an htb1-K119R mutant strain (JTB67-1). Gene groupings were created using wild-type RNAPII enrichment values. (C) Average distributions of differences between mutant and wild-type histone H3 enrichment grouped according to RNAPII enrichment in wild-type cells. The key below the graph illustrates the statistical significance of the differences for each group at 50 positions along the average gene. The rows of the key are color-coded according to the graph. Open squares denote p>0.01; light shading denotes 0.01>p>10exp-5 (one-sample t-tests; μ0 = 0). (PDF) [file pgen.1002822.s007.pdf]

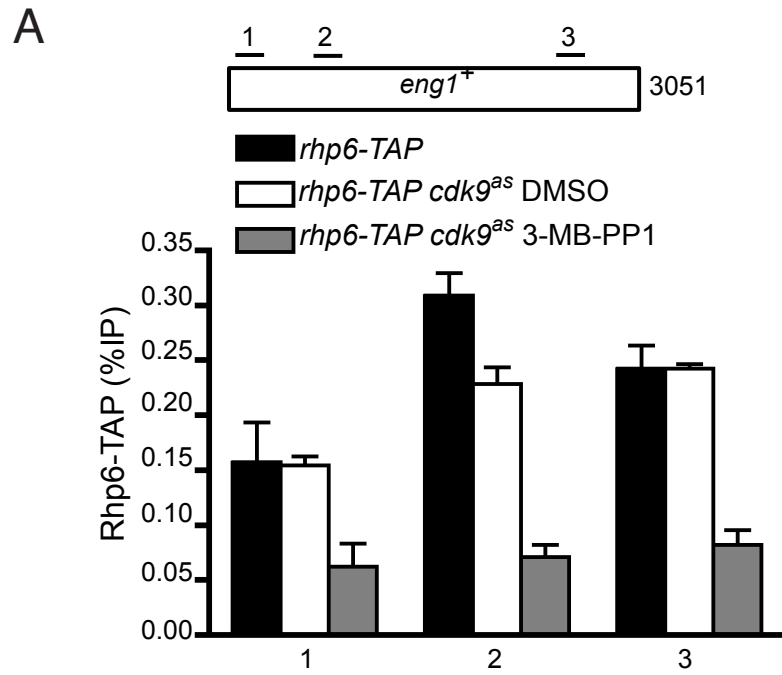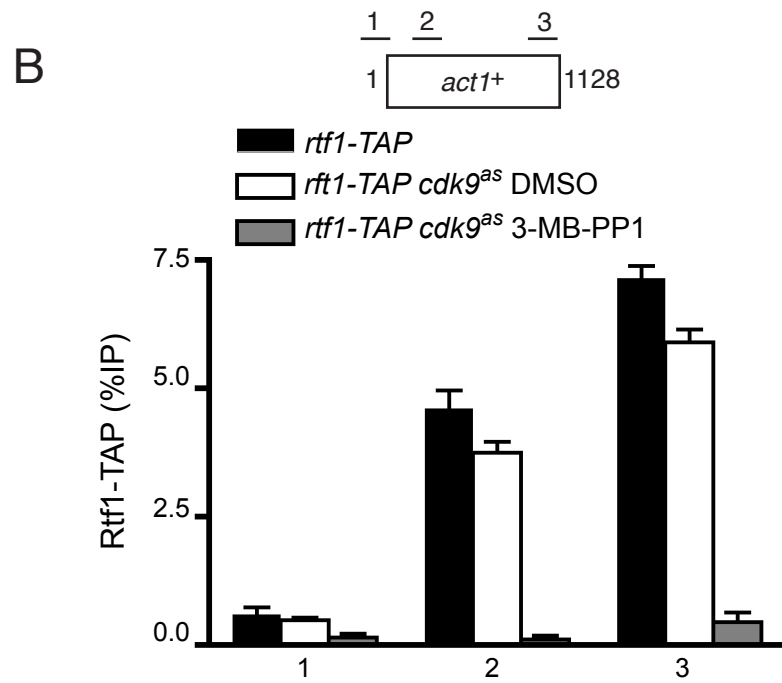

Supplement: Figure S3 — Chromatin association of Rhp6 and the PAF protein Rtf1 requires Cdk9 activity. (A) Rhp6-TAP occupancy was measured by ChIP in the indicated strains and quantified at the eng1+ gene by qPCR. Enrichment is plotted as percentage of the input signal for each primer pair. Positions of PCR primer pairs are indicated in the schematic at top. (B) Rtf1-TAP occupancy was measured by ChIP in the indicated strains and quantified at the act1+ gene by qPCR. Enrichment is plotted as percentage of the input signal for each primer pair. Positions of PCR primer pairs are indicated in the schematic at top. In all cases, treatment of the cdk9as strains was with either DMSO (-) or 20 µM 3-MB-PP1 for 3 hr prior to harvesting. Error bars denote standard deviations from three independent experiments. (PDF) [file pgen.1002822.s008.pdf]

A

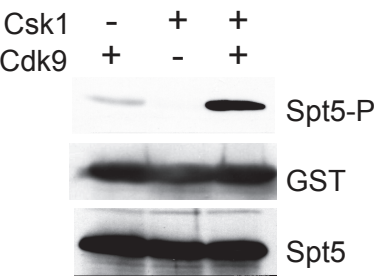

B

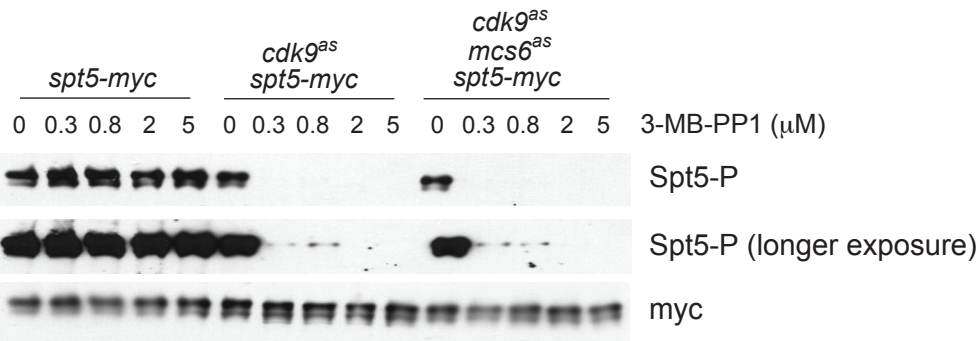

C

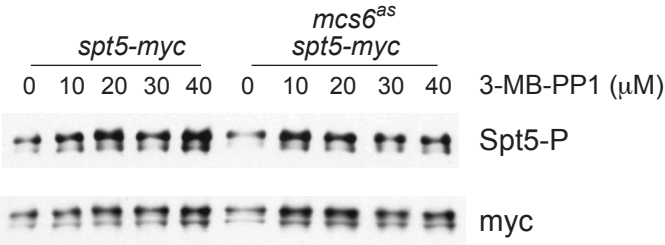

D

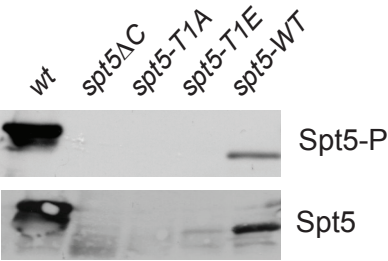

Supplement: Figure S4 — Validation of Spt5 as an exclusive Cdk9 target using a phospho-specific Spt5 antibody. (A) Kinase reactions containing recombinant GST-Spt5 substrate and indicated combinations of purified, recombinant Cdk9/Pch1 and Csk1 were analyzed by immunoblotting with: affinity-purified antibody specific for phosphorylated Spt5 (Spt5-P); crude serum specific for wild-type Spt5 CTD repeats, regardless of phosphorylation state (Spt5-CTD; see Text S1); or anti-GST antibody (GST). (B,C) Immunoblots of whole-cell extracts from indicated spt5-myc strains after treatment with increasing concentrations of 3-MB-PP1. Antibodies are indicated at right. (D) Both phospho-isoform- and pan-specific total Spt5 antibodies are specific for the wild-type nonapeptide repeat sequence of the Spt5 CTD. Whole-cell extracts from the indicated strains were probed with affinity-purified antibody (top, “Spt5-P”) or crude serum (bottom, “Spt5”) from a rabbit immunized with an Spt5-CTD phosphopeptide (see Materials and Methods). Spt5 antibodies discriminate against nonapeptides with substitutions at the Thr1 position, but not against unphosphorylated, wild-type Spt5-CTD (see Figure 3A), illustrating why phospho-site mutant proteins cannot be used as controls for specificity of phosphospecific antibodies. (PDF) [file pgen.1002822.s009.pdf]

A

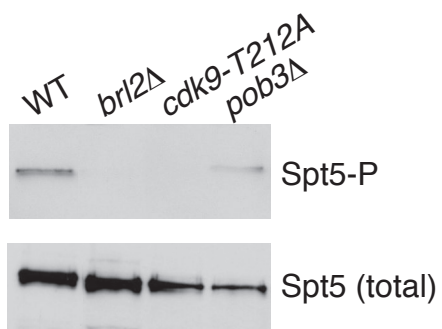

B

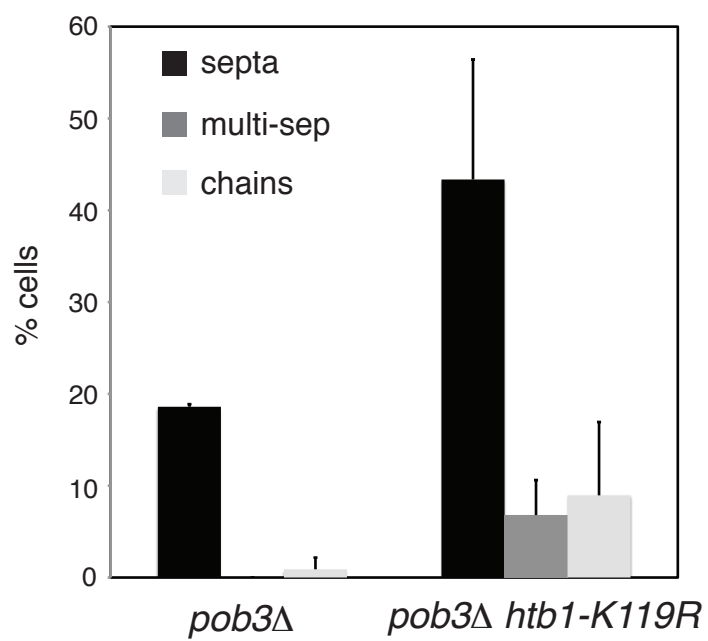

Supplement: Figure S5 — The FACT subunit Pob3 has functions distinct from those of H2Bub1. (A) Immunoblots of whole-cell extracts from wild-type (JTB204), brl2Δ (JTB331), cdk9-T212A (HD7-24), and pob3Δ (JTB281-1) strains using the indicated antibodies. (B) Quantification of abnormal septation in strains of indicated genotypes (JTB317 and JTB318, respectively). Error bars represent standard deviations from 2 independent experiments; at least 200 cells were counted in each. (PDF) [file pgen.1002822.s010.pdf]

A

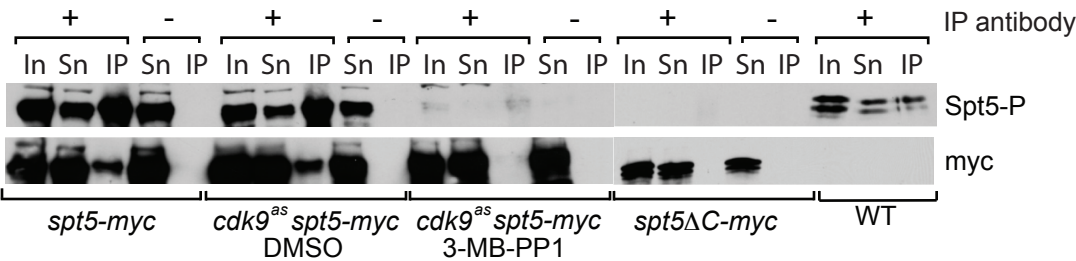

B

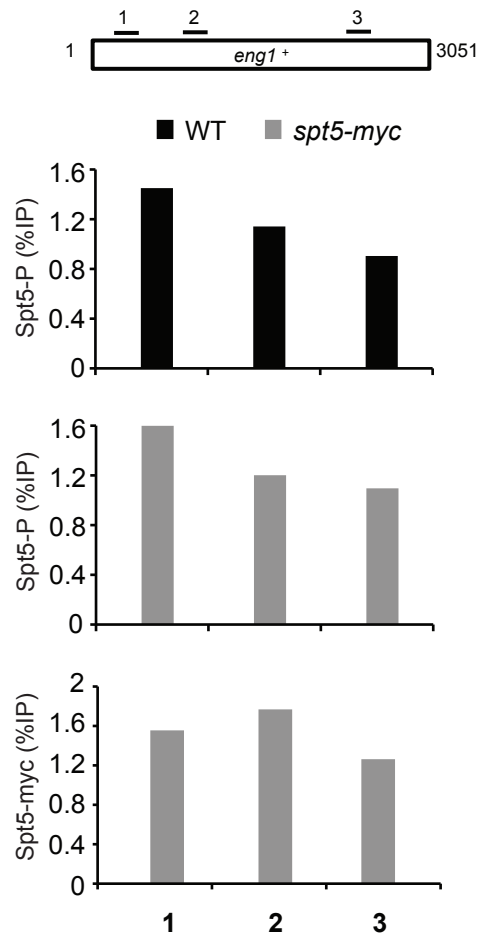

C

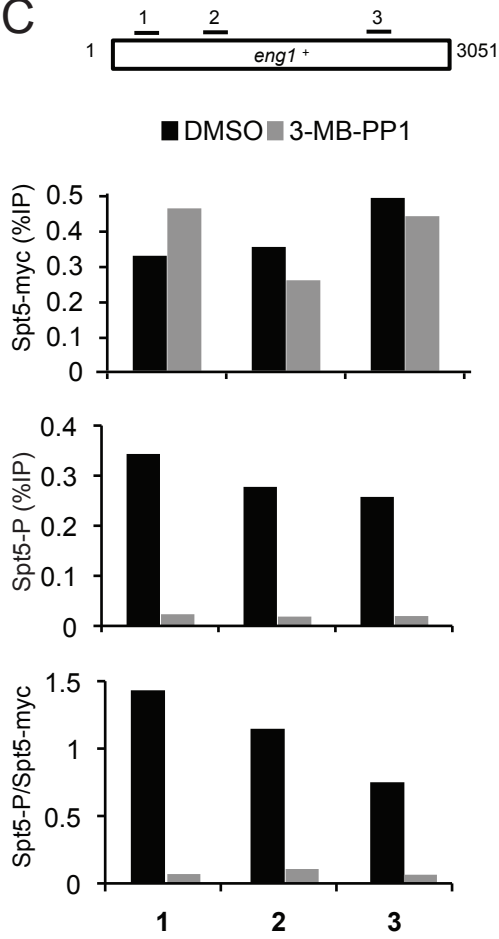

Supplement: Figure S6 — Anti-Spt5-P specifically immunoprecipitates Spt5-P from wild-type and spt5-myc strains. (A) Spt5-P immunoprecipitation from whole-cell extracts. Extracts from indicated strains (bottom) were incubated with or without anti-Spt5-P (top), and immunoprecipitates were analyzed by immunoblotting with antibodies indicated at right. The cdk9as strain was treated prior to extract preparation with either DMSO or 5 µM 3-MB-PP1, as indicated at bottom, for 20 min. For each treatment we analyzed 10% of input extract (“input”), 10% of supernatant after precipitation (“sup”) and 50% of immunoprecipitated material (“IP”). Antibodies are indicated at right. (B) ChIP of Spt5-P and Spt5-myc at eng1 +. Patterns of Spt5-P occupancy on eng1+ are similar in wild-type (“WT,” top; JS78) and spt5-myc (middle; CS111) strains. Distribution of total Spt5 on eng1+ was measured in the spt5-myc strain (bottom). (C) Spt5-myc (top) and Spt5-P (middle) occupancies were measured by ChIP in a cdk9as spt5-myc strain and quantified at indicated positions within eng1 + by qPCR. Cells were treated prior to lysis with either DMSO or 5 µM 3-MB-PP1 for 20 min. Enrichment is plotted as percentage of input signal for each primer pair. The ratios of Spt5-P/Spt-myc at each position are plotted at bottom. (PDF) [file pgen.1002822.s011.pdf]

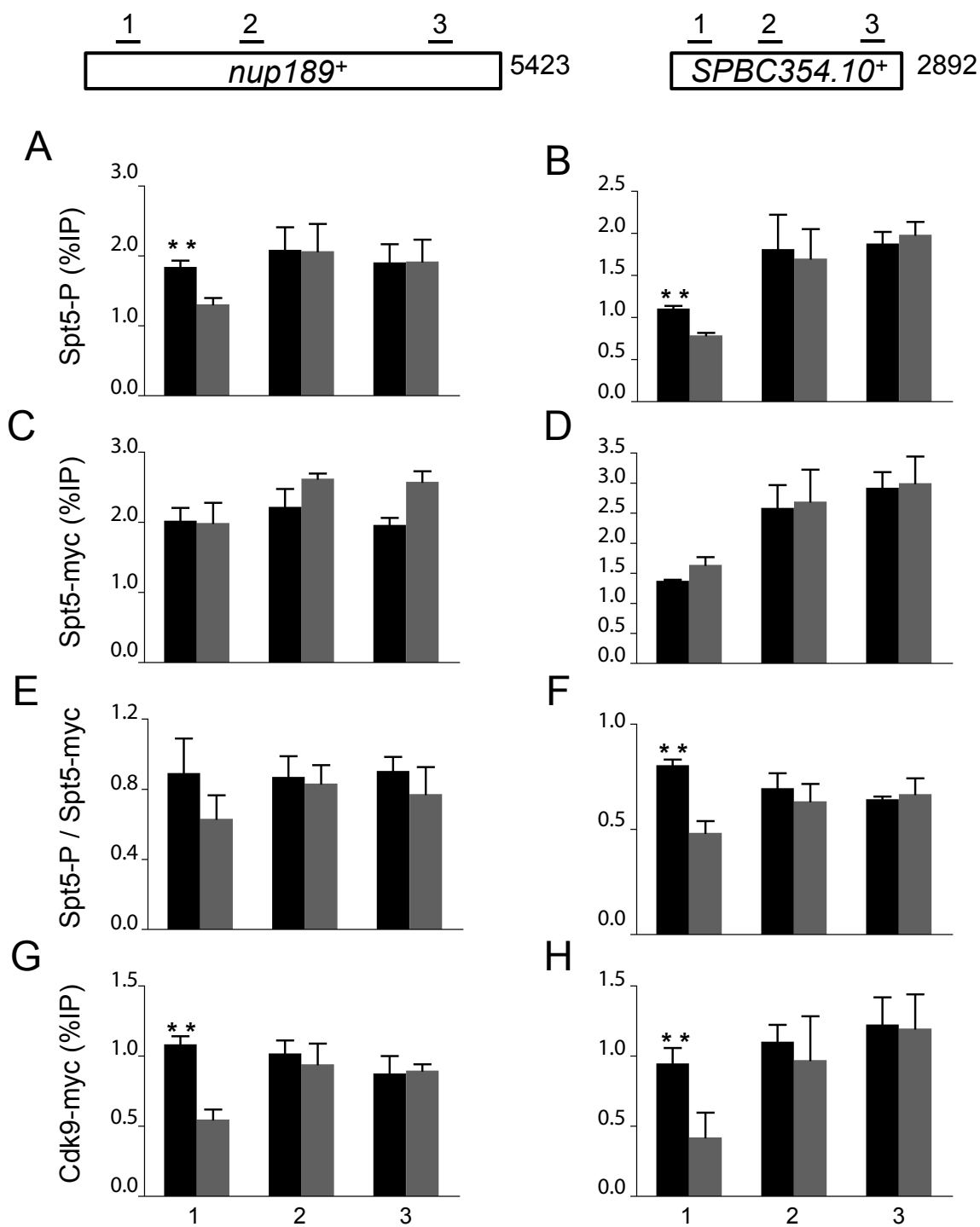

Supplement: Figure S7 — Impact of H2Bub1 on Spt5 phosphorylation and Cdk9 recruitment at nup189+ and SPB354.10+. (A,B) Spt5 phosphorylation was measured by ChIP using anti-Spt5-P in spt5-myc (MS265; black bars) and spt5-myc htb1-K119R (LV239; gray bars) strains and quantified at the indicated genes by qPCR. In all graphs of this figure the enrichment is plotted as percentage of the input signal for each primer pair, positions of PCR primer pairs within coding regions are indicated in the schematic at top, error bars denote standard deviations from 3 independent experiments and asterisks denote a significant difference between wild-type and mutant (“*” p<0.04, “**” p<0.02; unpaired t-test). (C,D) Spt5-myc occupancy was measured by ChIP as in C and D. (E,F) Spt5-P enrichment normalized to total Spt5-myc occupancy. (G,H) Cdk9-myc occupancy was measured by ChIP in cdk9-myc (MS264; black bars) and cdk9-myc htb1-K119R (KL259; gray bars) strains. (PDF) [file pgen.1002822.s012.pdf]

A

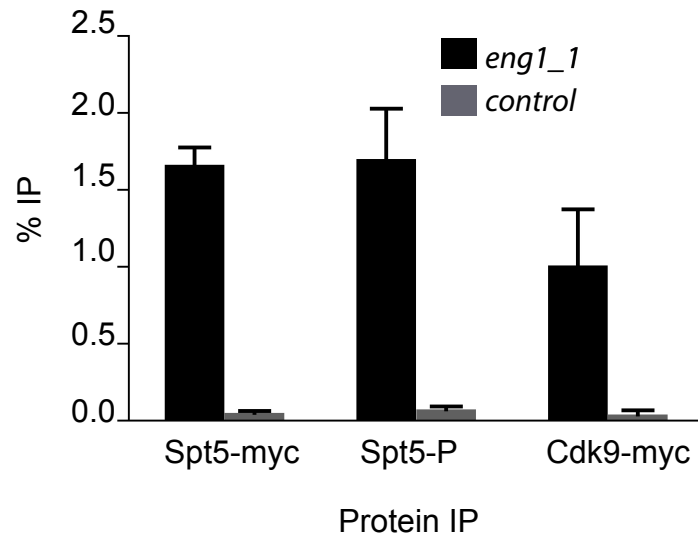

B

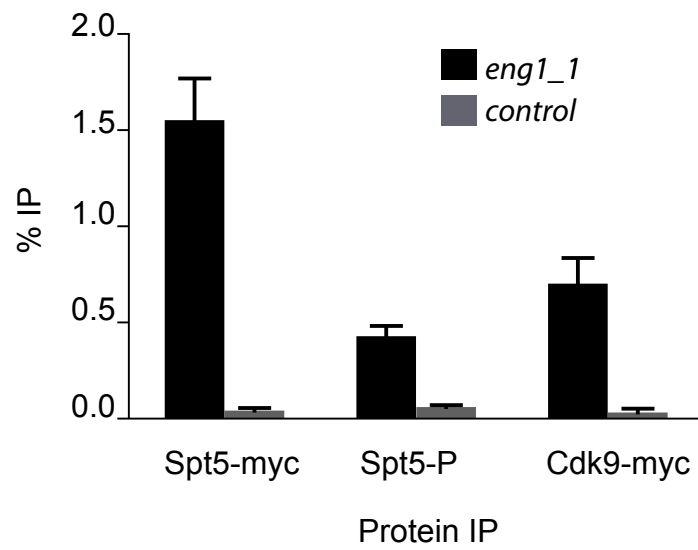

Supplement: Figure S8 — ChIP of Spt5-P, Spt5-myc, and Cdk9-myc at an intergenic sequence. (A) Occupancy of total Spt5, phospho-Spt5 and Cdk9 was measured by ChIP using anti-myc or anti-Spt5-P in wild-type strains (MS265 for Spt5-myc and Spt5-P IP; MS264 for Cdk9-myc IP), followed by qPCR using primers covering the 5′ end of eng1 (eng1-1) and an intergenic region as control (control). In all graphs of this figure the enrichment is plotted as percentage of the input signal for each primer pair and error bars denote standard deviations from 3 independent experiments. (B) Same experiment as in (A) in htb1-K119R strains (LV239 for Spt5-myc and Spt5-P IP; KL259 for Cdk9-myc IP). (PDF) [file pgen.1002822.s013.pdf]

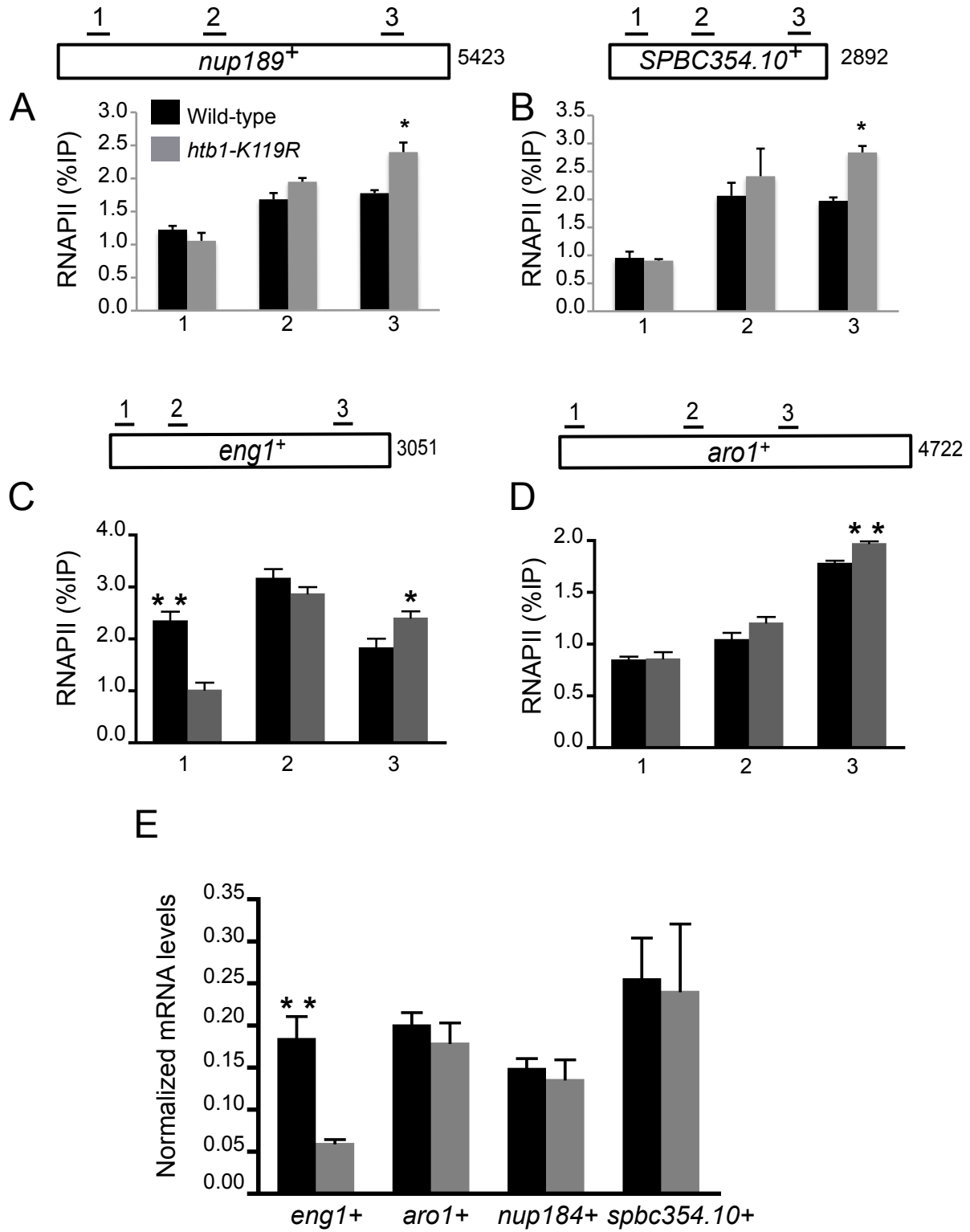

Supplement: Figure S9 — Impact of H2Bub1 on RNAPII occupancy and mRNA expression at individual genes. (A-D) RNAPII occupancy was measured by ChIP in wild-type (JTB62-1; black bars) and htb1-K119R (JTB67-1; gray bars) strains and quantified at the indicated genes by qPCR. Enrichment is plotted as percentage of the input signal for each primer pair, and positions of PCR primer pairs within coding regions are indicated in the schematic at top of each graph. (E) Levels of mRNA from the indicated genes were quantified by qRT-PCR and normalized to act1+. Throughout this figure error bars denote standard deviations from 3 independent experiments and asterisks denote a significant difference between wild-type and mutant (“*” p<0.04, “**” p<0.02; unpaired t-test). (PDF) [file pgen.1002822.s014.pdf]

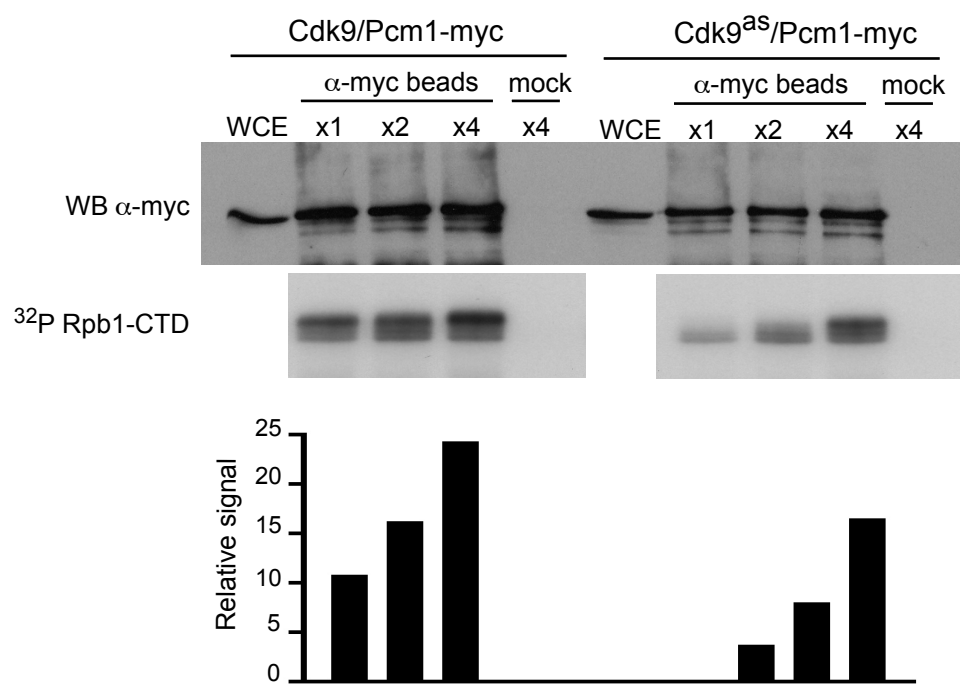

Supplement: Figure S10 — Kinase activity of Cdk9 and Cdk9as complexes purified from S. pombe. Lysates from pcm1-myc (CS145) or cdk9as pcm1-myc (CS165) strains were incubated either with anti-myc-bound protein G beads (α-myc-beads) to co-immunoprecipitate Cdk9 bound to Pcm1-myc or with protein G alone (mock). Input lysate amounts in each immunoprecipitation were varied as indicated. Immunoprecipitates were analyzed by immunoblotting (top panels) and for Rpb1-CTD kinase activity (middle panels). Phosphorylation signals were visualized by autoradiography and quantified with a phosphorimager (bottom). (PDF) [file pgen.1002822.s015.pdf]
