# Supplementary material for: A Positive Feedback Loop Links Opposing Functions of P-TEFb/Cdk9 and Histone H2B Ubiquitylation to Regulate Transcript Elongation in Fission Yeast
Source: PLoS Genet. 2012 Aug 2;8(8):e1002822. doi: 10.1371/journal.pgen.1002822 (PMC3410854; doi:10.1371/journal.pgen.1002822)
Supplement: Table S1 — RNAPII distributions at H2Bub1-stimulated genes in the htb1-K119R strain. (DOC) [file pgen.1002822.s016.doc]

**Table S1**. RNAPII distributions at H2Bub1-stimulated genes in the *htb1-K119R* strain.

| Gene name   | Systematic name | Description | RNAPII pattern |  | | --- | --- | --- | --- | | ytm1 | SPAC890.04c | microtubule-associated protein | A | | spo4 | SPBC21C3.18 | serine/threonine protein kinase Spo4 | B | | rpl16c.A | SPBC2G2.05 | 60S ribosomal protein L13/L16 | B | | rpa43 | SPBC3B9.07c | DNA-directed RNA polymerase I complex subunit Rpa43 | B | | rds1 | SPAC343.12 | conserved fungal protein | B | | ptr2 | SPBC13A2.04c | PTR family peptide transporter | B | |  | SPBP22H7.06 | nicotinamide riboside kinase | B | |  | SPAPB1E7.04c | chitinase | B | |  | SPBP8B7.05c | carbonic anhydrase | B | |  | SPAP8A3.07c | phospho-2-dehydro-3-deoxyheptonate aldolase | B | | hem2 | SPAC1805.06c | porphobilinogen synthase Hem2 | A | | fio1 | SPAC1F7.08 | iron transport multicopper oxidase | B | | cad1: hmt2 | SPBC2G5.06c | sulfide-quinone oxidoreductase | B | |  | SPAC977.12 | L-asparaginase | N/A | |  | SPBC947.04 | glycoprotein | B | |  | SPBC776.03 | homoserine dehydrogenase | B | |  | SPCC613.01 | membrane transporter | A | |  | SPCC622.12c | NADP-specific glutamate dehydrogenase | B | |  | SPAC4A8.10 | lipase | A | | hsr1 | SPAC3H1.11 | transcription factor | B | | chr1 | SPBC3E7.12c | chitin synthase regulatory factor | B | | abc3 | SPBC359.05 | ABC transporter family | C | |  | SPBC359.03c | amino acid permease family | A | |  | SPCC330.07c | membrane transporter | A | | eta2 | SPAC31G5.10 | Myb family | B | |  | SPAC29B12.10c | OPT oligopeptide transporter family | B | |  | SPBC25B2.09c | arginine-tRNA ligase | B | |  | SPAC23C11.09 | alanine-tRNA ligase | B | |  | SPAC1F12.07 | phosphoserine aminotransferase | B | |  | SPAC1952.09c | acetyl-CoA hydrolase | A | |  | SPCC1840.07c | calcineurin-like phosphoesterase | C | | adg3 | SPCC18.01c | beta-glucosidase | B | |  | SPAC17A2.04c | HSP chaperone complex subunit | C | |  | SPBC1773.15 | membrane transporter | A | |  | SPBC1683.05 | thiamine transporter | B | |  | SPAC12B10.04 | tubulin-tyrosine ligase | B | |  | SPBC1271.10c | membrane transporter | A | | dea2 | SPBC1198.02 | adenine deaminase Dea2 | B | | cys2 | SPBC106.17c | homoserine O-acetyltransferase | A | | urg1 | SPAC1002.19 | GTP cyclohydrolase | C | | ade5: ade8 | SPCC569.08c | glycinamide ribonucleotide transformylase | B | |
| --- | --- | --- | --- | --- | --- | --- | --- | --- | --- | --- | --- | --- | --- | --- | --- | --- | --- | --- | --- | --- | --- | --- | --- | --- | --- | --- | --- | --- | --- | --- | --- | --- | --- | --- | --- | --- | --- | --- | --- | --- | --- | --- | --- | --- | --- | --- | --- | --- | --- | --- | --- | --- | --- | --- | --- | --- | --- | --- | --- | --- | --- | --- | --- | --- | --- | --- | --- | --- | --- | --- | --- | --- | --- | --- | --- | --- | --- | --- | --- | --- | --- | --- | --- | --- | --- | --- | --- | --- | --- | --- | --- | --- | --- | --- | --- | --- | --- | --- | --- | --- | --- | --- | --- | --- | --- | --- | --- | --- | --- | --- | --- | --- | --- | --- | --- | --- | --- | --- | --- | --- | --- | --- | --- | --- | --- | --- | --- | --- | --- | --- | --- | --- | --- | --- | --- | --- | --- | --- | --- | --- | --- | --- | --- | --- | --- | --- | --- | --- | --- | --- | --- | --- | --- | --- | --- | --- | --- | --- | --- | --- | --- | --- | --- | --- | --- | --- | --- | --- |
| A: RNAPII occupancy is increased at the 3’ end relative to wild-type |
| B: RNAPII occupancy is decreased throughout the coding region relative to wild-type |
| C: RNAPII occupancy is unchanged relative to wild-type |
| N/A: not available |
