# Supplementary material for: A Positive Feedback Loop Links Opposing Functions of P-TEFb/Cdk9 and Histone H2B Ubiquitylation to Regulate Transcript Elongation in Fission Yeast
Source: PLoS Genet. 2012 Aug 2;8(8):e1002822. doi: 10.1371/journal.pgen.1002822 (PMC3410854; doi:10.1371/journal.pgen.1002822)
Supplement: Table S2 — S. pombe strains used in this study. (DOC) [file pgen.1002822.s017.doc]

**Table S2**. *S. pombe* strains used in this study.

| Name | Genotype | Source |
| --- | --- | --- |
| JTB62-1 | *h- ade6-M216 htb1-FLAG::kanMX6* | Tanny et al., 2007 |
| JTB67-1 | *h- ade6-M216 htb1-K119R-FLAG::kanMX6* | Tanny et al., 2007 |
| JTB325 | *cdk9-T212A::kanMX6 htb1-FLAG::kanMX6 ade6 his3? h?* | This study |
| JTB326 | *cdk9-T212A::kanMX6 htb1-K119R-FLAG::kanMX6 ade6 his3? h?* | This study |
| JTB331 | *h- brl2∆::hphMX4 ade6-M210* | This study |
| JTB377 | *lsk1∆::ura4^+^ htb1-FLAG::kanMX6 ura4-D18 leu1-32 ade6? h?* | This study |
| JTB333 | *lsk1∆::ura4^+^ htb1-K119R-FLAG::kanMX6 ura4-D18 leu1-32 ade6? h?* | This study |
| JTB335 | *cdk9-T212A::kanMX6 brl2∆::hphMX4 leu1-32 ura4-D18 his3? ade6* | This study |
| *lsk1∆* | *lsk1∆::ura4^+^ leu1-32 ura4-D18 h+* | Karagiannis et al, 2007b |
| *spt5-WT* | *spt5-WT(7)::ura4^+^ ade6-M210 leu1-32 ura4-D18 his3-D1 h+* | Schneider et al, 2010 |
| *spt5-T1A* | *spt5-T1A(7)::ura4^+^ ade6-M210 leu1-32 ura4-D18 his3-D1 h+* | Schneider et al, 2010 |
| *spt5-T1E* | *spt5-T1E(7)::ura4^+^ ade6-M210 leu1-32 ura4-D18 his3-D1 h+* | Schneider et al, 2010 |
| *spt5∆C* | *spt5∆C::ura4^+^ ade6-M210 leu1-32 ura4-D18 his3-D1 h+* | Schneider et al, 2010 |
| JTB378 | *spt5-WT(7)::ura4^+^ htb1-FLAG::kanMX6 ade6 leu1-32 ura4-D18 his3? h+* | This study |
| JTB351 | *spt5-WT(7)::ura4^+^ htb1-K119R-FLAG::kanMX6 ade6 leu1-32 ura4-D18 his3-D1 h?* | This study |
| JTB379 | *spt5-T1A(7)::ura4^+^ htb1-FLAG::kanMX6 ade6 leu1-32 ura4-D18 his3? h+* | This study |
| JTB353 | *spt5-T1A(7)::ura4^+^ htb1-K119R-FLAG::kanMX6 ade6 leu1-32 ura4-D18 his3-D1 h?* | This study |
| JTB380 | *spt5-T1E(7)::ura4^+^ htb1-FLAG::kanMX6 ade6 leu1-32 ura4-D18 his3? h?* | This study |
| JTB355 | *spt5-T1E(7)::ura4^+^ htb1-K119R-FLAG::kanMX6 ade6 leu1-32 ura4-D18 his3-D1 h?* | This study |
| JS78 | *leu1-32 ura4-D18 his3-D1 ade6-M210 h+* | J Hurwitz |
| LV7 | *cdk9^as^::kanMX6 leu1-32 ura4-D18 his3-D1 ade6-M210 h+* | Viladevall et al., 2009 |
| LV77 | *mcs6^as^::kanMX6 leu1-32 ura4-D18 his3-D1 ade6-M216 h-* | Viladevall et al., 2009 |
| LV46 | *cdk9^as^::kanMX6 mcs6^as^:: kanMX4 leu1-32 ura4-D18 his3-D1 ade6-M21X h-* | This study |
| CS111 | *spt5-13myc::kanMX6 leu1-32 ura4-D18 his3-D1 ade6-M210 h+* | Viladevall et al., 2009 |
| CS155 | *mcs6^as^::kanMX6 spt5-13myc::kanMX6 leu1-32 ura4-D18 his3-D1 ade6-M21X h-* | Viladevall et al., 2009 |
| CS112 | *cdk9^as^::kanMX6 spt5-13myc::kanMX6 leu1-32 ura4-D18 his3-D1 ade6-M21X h+* | Viladevall et al., 2009 |
| JS207 | *mcs6-S165A-HA3::kanMX leu1-32 ura4-D18 his3-D1 ade6-M216 h-* | J Saiz |
| HD7-24 | *cdk9-T212A::kanMX6 leu1-32 ura4-D18 his3-D1 ade6-M210 h+* | H Du |
| *lsc1∆* | *lsc1∆*::*kanMX6 h-* | M Shimanuki |
| JS80 | *csk1∆::ura+ leu1-32 ura4-D18 his3-D1 ade6-M210 h+* | J Saiz |
| CS159 | *lsk1^as^::kanMX6 spt5-13myc::kanMX6 leu1-32 ura4-D18 his3-D1 ade6-M216 h-* | This study |
| LV239 | *htb1-K119R-FLAG*::*kanMX6 spt5-13myc::kanMX6 leu1-32 ura4-D18 his3-D1 ade6-M21X h?* | This study |
| LV167 | *spt5∆C-myc::kanMX6 leu1-32 ura4-D18 his3-D1 ade6-M210 h-* | This study |
| HD6-51 | *cdk9-13myc::kanMX6 leu1-32 ura4-D18 his3-D1 ade6-M210 h+* | Pei et al. 2006 |
| KL259 | *htb1-K119R-FLAG*::*kanMX6 cdk9-13myc::kanMX6 leu1-32 ura4-D18 his3-D1 ade6-M210 h?* | This study |
| LV193 | *cdk9^as^::kanMX6 htb1-K119R-FLAG*::*kanMX6 leu1-32 ura4-D18 his3-D1 ade6-M210 h?* | This study |
| JTB297 | *ubp8∆::hphMX6 ade6-M216 h-* | This study |
| JTB336 | *cdk9-T212A::kanMX6 upb8∆::hphMX6 leu1-32 ura4-D14 his3-D1 ade6-M21X h?* | This study |
| JTB204 | *h- ade6-M216* | Tanny et al.  2007 |
| JTB202 | *h- ade6-M216 rtf1-TAP::kanMX6* | This study |
| JTB80-2 | *h- ade6-M216 set1∆::kanMX6* | Tanny et al.  2007 |
| JTB86 | *h- ade6-M216 htb1-K119R::kanMX6* | Tanny et al.  2007 |
| JTB350 | *spt5-WT(7)::ura4^+^ set1∆::kanMX6 ade6-M210 leu1-32 ura4-D18 his3-D1 h+* | This study |
| JTB352 | *spt5-T1A(7)::ura4^+^ set1∆::kanMX6 ade6-M210 leu1-32 ura4-D18 his3-D1 h+* | This study |
| JTB354 | *spt5-T1E(7)::ura4^+^ set1∆::kanMX6 ade6-M210 leu1-32 ura4-D18 his3-D1 h+* | This study |
| JTB418 | *spt5-T1A(7)::ura4^+^ set2∆::hphMX6 ade6-M210 leu1-32 ura4-D18 his3-D1 h?* | This study |
| JTB428 | *cdk9-T212A::kanMX6 set1∆::kanMX6 leu1-32 ura4-D18 his3-D1 ade6-M210 h?* | This study |
| JTB392 | *cdk9^as^::kanMX6 rtf1-TAP::kanMX6 ade6 h?* | This study |
| LV252 | *cdk9-T212A::kanMX6 htb1-K119R-FLAG*::*kanMX6 leu1-32 ura4-D18 his3-D1 ade6-M210 h?* | This study |
| LV256 | *cdk9-T212E::kanMX6 htb1-K119R-FLAG*::*kanMX6 leu1-32 ura4-D18 his3-D1 ade6-M210 h?* | This study |
| MS264 | *cdk9::13myc::kanMX6 htb1-FLAG::natMX6 leu1-32 ura4-D18 his3-D1 ade6-M21X h+* | This study |
| MS265 | *spt5::13myc::kanMX6 htb1-FLAG::natMX6 leu1-32 ura4-D18 his3-D1 ade6-M21X h+* | This study |
| MS249 | *cdk9^as^::kanMX6 htb1-FLAG::natMX6 leu1-32 ade6-M21X h-* | This study |
| MS250 | *htb1-FLAG::natMX6 ade6-M210 h-* | This study |
| MS260 | *rpb1-16:: natMX6 leu1-32 ura4-D18 his3-D1 ade6-M210 h+* | This study |
| MS261 | *rpb1-S2A::natMX6 leu1-32 ura4-D18 his3-D1 ade6-M210 h+* | This study |
| MS256 | *rpb1-16::natMX6 htb1-K119R-FLAG::kanMX6 ade6-M216 h-* | This study |
| MS257 | *rpb1-S2A::natMX6 htb1-K119R-FLAG::kanMX6 ade6-M216 h-* | This study |
| MS272 | *spt5-T1A(7)::ura4^+^ rpb1-S2A::natMX6 htb1-K119R-FLAG::kanMX6 ade6-M210 ura4-D18 h-* | This study |
| MS259 | *cdk9^as^::kanMX6 htb1-K119R-FLAG::kanMX6 ^+^ rpb1-S2A:: natMX6 leu1-32 ura4-D18 his3-D1 ade6-M210 h+* | This study |
| KL289 | *cdk9^as^::kanMX6 htb1-K119R-FLAG::kanMX6 ^+^ spt5-WT(7)::ura4^+^ ura4-D18 ade6-M21?* | This study |
| KL291 | *cdk9^as^::kanMX6 htb1-K119R-FLAG::kanMX6 ^+^ spt5-T1A(7)::ura4^+^ ura4-D18 his? leu1? ade6-M21?* | This study |
| KL293 | *cdk9^as^::kanMX6 htb1-K119R-FLAG::kanMX6 ^+^ spt5-T1E(7)::ura4^+^ ura4-D18 his? leu1? ade6-M21?* | This study |
| JTB281-1 | *h- ade6-M216 pob3∆::hphMX6* | This study |
| JTB317 | *pob3∆::hphMX6 htb1-FLAG::kanMX6 ade6 h?* | This study |
| JTB318 | *pob3∆::hphMX6 htb1-K119R-FLAG::kanMX6 ade6 h?* | This study |
| CS145 | *pcm1-13myc::kanMX6 leu1-32 ura4-D18 his3-D1 ade6-M216 h-* | St Amour et al, 2012 |
| CS165 | *cdk9^as^:: kanMX6 pcm1-13myc::kanMX6 leu1-32 ura4-D18 his3-D1 ade6-M216 h-* | Viladevall et al, 2009 |
| MS337 | *rhp6-TAP::kanMX6 cdk9^as^:: natMX6 leu1-32 ura4-D18 his3-D1 ade6-M216 h-* | This study |
