# Supplementary material for: A Positive Feedback Loop Links Opposing Functions of P-TEFb/Cdk9 and Histone H2B Ubiquitylation to Regulate Transcript Elongation in Fission Yeast
Source: PLoS Genet. 2012 Aug 2;8(8):e1002822. doi: 10.1371/journal.pgen.1002822 (PMC3410854; doi:10.1371/journal.pgen.1002822)
Supplement: Text S1 — Supplemental materials and methods. (DOC) [file pgen.1002822.s018.doc]

**Supplemental Material for Sansó, Lee, Viladevall, et al., “A positive feedback loop links opposing functions of P-TEFb/Cdk9 and histone H2B ubiquitylation to regulate transcript elongation in fission yeast”**

Supplemental Materials and Methods

Supplemental Tables S1-S2

Supplemental Datasets S1-S5 (ChIP-chip of H2Bub1, RNAPII, H3)

**Supplemental Materials and Methods**

**Microarray analysis**. *S. pombe* whole-genome tiling arrays containing 4x44K probes were purchased from Agilent Technologies. Arrays were scanned on an Axon GenePix 4000B scanner. Analyses were carried out as described previously [1]. For H2Bub1 ChIP-chip experiments, enrichment was calculated as the ratio of H2Bub1 to FLAG signals (to normalize for total levels of H2B-FLAG). For RNAPII and histone H3 experiments, occupancy was calculated as the ratio of IP signal to input. Each experiment was carried out in duplicate. BED files of the normalized ChIP-chip data are included as Datasets S1-S5.

**Mapping data to average genes**. To interpolate between probes, a standard Gaussian filter (SD = 200 bp) was applied twice to the data as described previously [1] to generate a value each 10 bp. These ‘‘smoothed’’ data were used to calculate the average signal on the complete length of each gene. Only genes with two identified UTRs were then binned into groups as described in the figure legend. The non-smoothed data were mapped on the 5’ and 3’ UTR boundaries into 50 bp windows for each half-gene and adjacent half-intergenic regions. A sliding window of 300 bp was then applied to the ratios.

**Biochemical methods**. Purification of recombinant Cdk9/Pch1, Csk1, and GST-Spt5, and assay conditions for detection of Cdk9 activity in vitro, have been described previously [2].

**Antibodies**. The anti-Spt5 antibody used for immunoblotting was crude serum from a rabbit immunized with the Thr1-phosphorylated phosphopeptide; it is specific for the wild-type nonapeptide repeat of the Spt5-CTD but does not distinguish phosphorylated from unphosphorylated forms (Figures S4A and S4D). The H2Bub1-specific antibody used in ChIP was a gift from N. Minsky and M. Oren (Weizmann Institute, Israel). FLAG antibody was from Sigma. Monoclonal antibodies 8WG16, recognizing the RNAPII CTD, and 9E10, recognizing the Myc epitope, were from Covance. The H2Bub, H2B and K3K4me2 antibodies used for immunoblotting were from Millipore. H3K36me3 and H3 antibodies were from Abcam.

**Immunoprecipitation.** Immunoprecipitations were performed with Spt5-P and 9E10 antibodies bound to Protein G-Sepharose (GE Healthcare), or Protein G-Sepharose alone as a negative control. Beads were washed three times with 10 mM Hepes (pH 7.4), 150 mM NaCl, 0.1% Triton X-100 and twice with 10 mM Hepes (pH 7.4), 150 mM NaCl. The IPs (50%), supernatants (2.5%) and inputs (2.5%) were subjected to immunoblot analysis.

**RNA isolation and qRT-PCR**.

For qPCR analysis RNA from 50 ml of cells, exponentially growing in rich media (OD_600_≈0.5), was extracted with phenol and precipitated in ethanol. First-strand cDNA was synthesized from 2 µg total RNA, using oligo-d(T) from Super-Script First-Strand Synthesis kit (Invitrogen), and was quantified with a Stratagene MX3000P instrument with SYBRgreen ROX qPCR mastermix (Qiagen).

**Kinase assays**. Kinase assays were performed as described elsewhere [3].

**Supplemental References**

1. Rufiange A, Jacques PE, Bhat W, Robert F, Nourani A (2007) Genome-wide replication-independent histone H3 exchange occurs predominantly at promoters and implicates H3 K56 acetylation and Asf1. Mol Cell 27: 393-405.

2. Pei Y, Du H, Singer J, St Amour C, Granitto S, et al. (2006) Cyclin-dependent kinase 9 (Cdk9) of fission yeast is activated by the CDK-activating kinase Csk1, overlaps functionally with the TFIIH-associated kinase Mcs6, and associates with the mRNA cap methyltransferase Pcm1 in vivo. Mol Cell Biol 26: 777-788.

3. St. Amour CV, Sansó M., Bösken CA, Lee KM, Larochelle S, et al. (2012) Separate domains of fission yeast Cdk9 (P-TEFb) are required for capping enzyme recruitment and primed (Ser7-phosphorylated) Rpb1 carboxyl-terminal domain substrate recognition. Mol Cell Biol 32: 2372-2383.
